# Low-threshold interlayer exciton multiplication in twisted transition metal dichalcogenides heterobilayers

**DOI:** 10.1038/s41377-026-02193-w

**Published:** 2026-02-10

**Authors:** Pengzhi Wang, Gan Wang, Chenhao Wang, Qi Wei, Yuxuan Chen, Qi Liu, Luwei Zhou, Hui Ren, Xiang Zhang, Jiangbo Peng, Leyi Zhao, Tao-Yuan Du, Mingjie Li

**Affiliations:** 1 Department of Applied Physics, The Hong Kong Polytechnic University, Hung Hom, Kowloon China; 2 School of Mathematics and Physics, China University of Geosciences, Wuhan, 430074 China; 3 Shenzhen Research Institute, The Hong Kong Polytechnic University, Shenzhen, Guangdong 518057 China; 4 Photonics Research Institute, The Hong Kong Polytechnic University, Hung Hom, Kowloon China

**Keywords:** Single photons and quantum effects, Optical materials and structures

## Abstract

Multiple exciton generation (MEG) from single-photon absorption can enhance quantum efficiencies in light emission and energy conversion. However, its practical application is limited by high photon-energy thresholds, large material bandgaps, and rapid exciton recombination. Here, we report the observation of interlayer exciton multiplication (IXM) in twisted van der Waals heterobilayers, where the process threshold is as low as twice the type-II bandgap and the resulting multiple interlayer excitons (IXs) exhibit nanosecond-scale lifetimes. These properties directly address the core challenges of MEG, leading to a significant boost in both interlayer exciton emission and photocurrent quantum efficiency. Theoretical calculations confirm the experimental results, revealing that low-threshold IXM is facilitated by interlayer hot-carrier scattering and is accompanied by attractive IX interactions, even at larger twist angles where momentum mismatch is significant. Twist-angle-induced momentum mismatches have a minimal impact on the IXM threshold due to strong hot-carrier interlayer transfer and efficient intervalley impact ionization. The IXM efficiency reaches ~90% in small-twist-angle heterobilayers but decreases at larger angles and higher pump photon energies, a trend attributed to reduced interlayer hot-carrier Coulomb scattering. Furthermore, we demonstrate photocurrent multiplication driven by IXM. Applied electric fields further lower the IXM threshold and increase the IX yield, enabling a twofold enhancement in quantum efficiency and a fourfold increase in responsivity in a self-powered heterobilayer photodiode. Our results establish IXM as a promising mechanism for high-efficiency carrier-multiplication optoelectronics and provide insights into the many-body physics of stable multiple excitons.

## Abstract (web summary)

Low-threshold exciton multiplication in twisted heterobilayers features long-lived interlayer excitons, enhancing emission and photocurrent quantum efficiencies.

## Graphical abstract

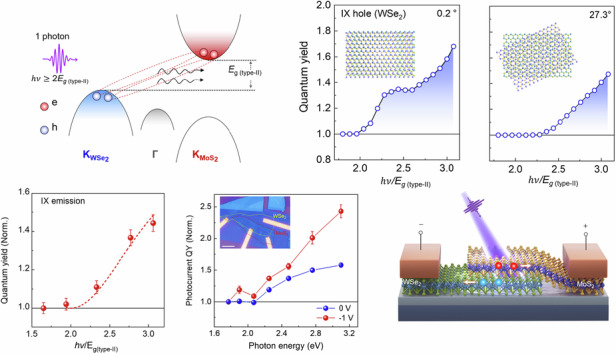

## Introduction

Single-photon-driven multiple exciton generation (MEG) is a process in which the absorption of a single high-energy photon generates two or more electron-hole pairs. Instead of dissipating its excess kinetic energy as heat, the initially excited “hot” carrier can promote an additional electron across the bandgap via impact ionization. MEG holds great promise for breaking the Shockley-Queisser limit by enhancing the quantum efficiency of optoelectronic devices^1–4^. Ideally, the threshold photon energy (*hν*_th_) for MEG is twice the material bandgap (*E*_g_). However, in practice, energy and momentum conservation constraints lead to low efficiencies and high thresholds (${h\nu }_{{\rm{th}}}$ ≥ 4${E}_{{\rm{g}}}$) for carrier multiplication in bulk semiconductors like Si and Ge^5–7^. While enhanced MEG has been demonstrated in quantum-confined systems such as PbSe, PbS^8–11^, Si^12^, Ge^13^ nanocrystals (NCs), as well as in few-layered 2D MoTe_2_^14,15^, their utility is hampered by the ultrafast Auger recombination of multiple excitons (e.g., within around 5 ps in few-layer MoTe_2_^14,15^) and large ${E}_{{\rm{g}}}$ (e.g., halide perovskite NCs^16^). Consequently, the development of efficient MEG materials that combine a low threshold, a tunable narrow bandgap, and slow multiple exciton recombination remains an outstanding challenge.

Two-dimensional van der Waals (vdW) heterobilayers, composed of different transition metal dichalcogenide (TMDC) monolayers, offer strong light-matter interactions and electrical and twist-angle tunability, enabling diverse phenomena in optoelectronics and quantum photonics^17–21^. A key feature of type-II heterobilayers is the formation of interlayer excitons (IXs), where electrons and holes are spatially separated across the two layers and bound across the staggered band structure^22–24^. This type-II alignment allows the interlayer bandgap (${E}_{{\rm{g}}\left({\rm{type}}-{\rm{II}}\right)}$) to be tuned from the visible range of the constituent monolayers into the near-infrared, with further electrical tunability^25,26^. Despite this potential, single-photon-driven generation of multiple interlayer excitons and their subsequent interactions have not been explored in twisted 2D vdW heterobilayers.

In this work, we report the observation of low-threshold interlayer exciton multiplication (IXM) in twisted type-II MoS_2_/WSe_2_ heterobilayers, yielding long-lived multiple IXs. This process leads to enhanced interlayer exciton emission and photocurrent quantum efficiency at room temperature. We find an IXM threshold energy as low as twice ${E}_{{\rm{g}}\left({\rm{type}}-{\rm{II}}\right)}$, approaching the fundamental energy conservation limit, which persists across all twist angles despite momentum mismatch. This is enabled by efficient hot-carrier interlayer transfer. Notably, the IXM efficiency reaches ~ 90% in small-twist-angle heterobilayers, where shorter attractive IX interaction distances facilitate the process, and gradually decreases with increasing twist angle due to reduced Coulomb interactions. Our experimental findings are well supported by theoretical modeling based on a tight-binding approach. Finally, we demonstrate IXM-driven photocurrent multiplication in a self-powered MoS_2_/WSe_2_ heterobilayer photodiode, achieving a threshold as low as 1.8${E}_{{\rm{g}}\left({\rm{type}}-{\rm{II}}\right)}$.

## Results

### Interlayer exciton emission multiplication

**Fig. 1:**
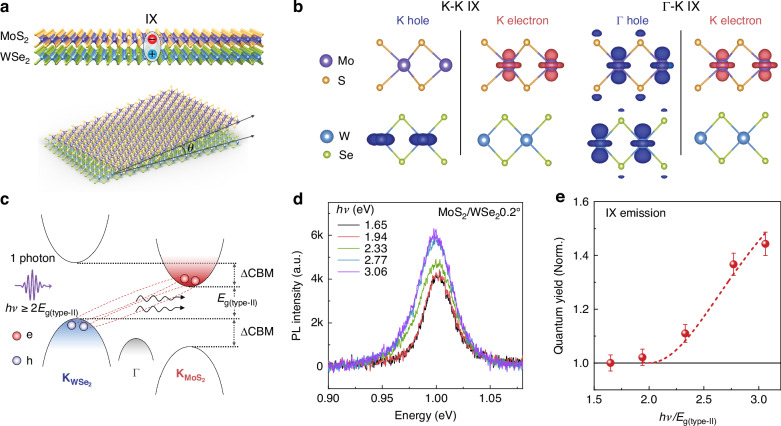
Multiple IX emission in a MoS_2_/WSe_2_ heterobilayer. **a** Schematic of MoS_2_/WSe_2_ heterobilayer showing an IX formed across the interface (upper) and twist angle of *θ* between two monolayers (lower). **b** DFT-calculated distribution of hole (left) and electron (right) states in a 0° aligned heterobilayer at the K and Γ valleys. **c** Energy diagram illustrating type-II band alignment with multiple IX emission process. **d** Infrared PL emission from the 0.2° heterobilayer at room temperature under different incident photon energies at a constant absorbed photon density of 1.8 × 10^12 ^cm^−2^. **e** IX PL quantum yield as a function of pump photon energy (normalized to ${E}_{g\left({\rm{type}}-{\rm{II}}\right)}$ of 1 eV). The dashed line is a fit using a MEG model (Supplementary Note 1) to extract IXM efficiency ${\eta }_{{\rm{IXM}}}$. Error bars represent the uncertainty in PL intensity determination

Figure 1a illustrates a twisted MoS_2_/WSe_2_ heterobilayer, where IXs form with electrons in MoS_2_ and holes in WSe_2_. The twist angle of each heterobilayer is determined using second-harmonic generation microscopy (see Supplementary Fig. 1 and “Methods” for details)^27–29^. Raman spectroscopy confirms strong interlayer coupling (Supplementary Note 2 and Supplementary Fig. 2a), and significant photoluminescence (PL) quenching of the constituent monolayers indicates efficient charge separation driven by the type-II band alignment (Supplementary Fig. 2b).

The calculated momentum-space structure of the heterobilayer is shown in Fig. 1b. The electron and hole states at the K valleys are localized within their respective layers (left panel), forming the dominant K-K IXs. In contrast, the hybrid hole state at the Γ point is distributed across both layers (right panel), giving rise to Γ-K IXs, consistent with previous reports^30,31^. The twist angle has only a minor effect on these charge-state distributions (Supplementary Fig. 3 and Supplementary Table 1). Figure 1c shows the schematic band alignment with conduction band minimum (CBM) and valence band maximum (VBM) offsets of ΔCBM = 0.76 eV and ΔVBM = 0.83 eV (ref. ^32^). From an energy conservation perspective, if the band-edge offset energy can be utilized as excess energy for impact ionization, it becomes possible to drive IXM when this energy exceeds ${E}_{{\rm{g}}\left({\rm{type}}-{\rm{II}}\right)}$.

To study IXM, we first calibrated the absorbed photon density using steady-state absorption spectra of the monolayers and heterobilayers (Supplementary Note 2 and Supplementary Fig. 4). In the near-infrared region, we observe prominent emission around 1.0 eV from K-K IXs at room temperature in the 0.2° heterobilayer (Fig. 1d). This confirms a type-II bandgap of ${E}_{{\rm{g}}\left({\rm{type}}-{\rm{II}}\right)}$ = 1.0 ± 0.1 eV, consistent with calculations based on monolayer bandgaps and the band offset. The PL intensity increases with pump photon energy. The PL quantum yield (QY) of the IX emission remains constant for $h\nu$ < 2${E}_{{\rm{g}}\left({\rm{type}}-{\rm{II}}\right)}$ but shows a significant increase once this threshold is exceeded (Fig. 1e). This clear threshold behavior signals the onset of IXM, with a fitted IXM efficiency (${\eta }_{{\rm{IXM}}}$) of 90 ± 5% (see the dashed-line fitting model in Supplementary Note 1). In heterobilayers with near-zero twist angles, the close alignment of the monolayer Brillouin zones^33^ allows IXs near the K-valley to exhibit nearly direct optical transitions, making them highly optically active.

### Long-lived multiple IXs

**Fig. 2:**
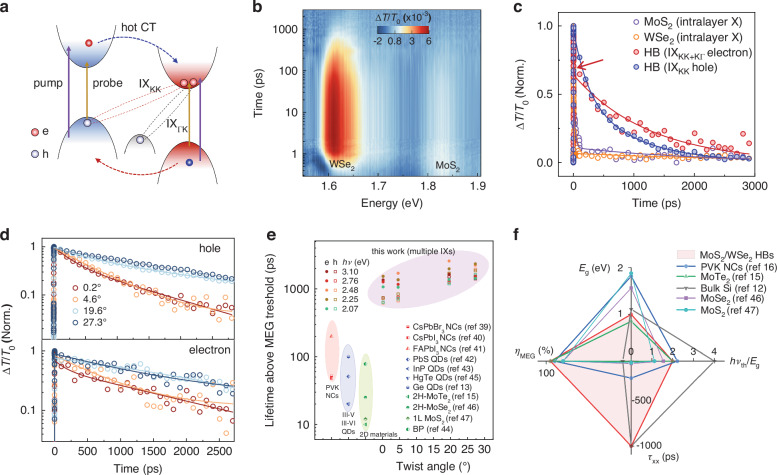
Photocarrier dynamics in twisted MoS_2_/WSe_2_ heterobilayers. **a** Schematic of the pump−probe configuration showing the individual K points of both materials and the hybridized Γ point, with probe energy tuned to the band-edge transitions of MoS_2_ and WSe_2_, respectively. The arrows illustrate the hot carrier transfer (CT) pathways for K-K and K-Γ IX formation. **b** 2D color plot of TA spectra of 0.2° heterobilayer following 2.48 eV photoexcitation. **c** PB dynamics at the MoS_2_ and WSe_2_ band edges for individual monolayers and the 0.2° heterobilayer under 2.48 eV excitation. **d** Twist-angle dependence of hole (top) and electron (bottom) population dynamics for IX states, with solid lines representing exponential fits. **e** Recombination lifetimes of IX electrons (solid circles) and holes (hollow squares) as a function of twist angle for excitation above the multiplication threshold, compared with reported multiple exciton lifetimes in other materials^13,15,39–47^. Complete TA dynamics and fitting parameters are provided in Supplementary Fig. 8 and Tables S2–S5. **f** Comparison of key MEG parameters between MoS_2_/WSe_2_ heterobilayers (HBs) and representative materials^12^^,15^^,16^^,^^46^^,47^. PVK perovskite, QD quantum dot, BP black phosphorus

To investigate the dynamics of multiple IXs and probe the IXM process, we used pump-probe transient absorption (TA) microscopy to monitor the population kinetics (Fig. 2a and Supplementary Fig. 5). The differential transmittance, $\Delta T/{T}_{0}$, is proportional to the total occupation of the electron and hole states involved in the probe beam transitions, [$\mathop{\sum}\nolimits_{i}\left({n}_{i}^{e}+{n}_{i}^{h}\right)$]. Figure 2b presents the 2D TA spectra for the 0.2° heterobilayer, revealing two distinct photobleaching (PB) bands at 1.81 eV and 1.61 eV, which correspond to the band-edge transitions of MoS_2_ and WSe_2_, respectively (see Supplementary Fig. 6 for other twist angles). The persistence of these PB signals—with recombination lifetimes two orders of magnitude longer than those of intralayer excitons—confirms efficient charge separation and the formation of stable IXs^23,34^ (Fig. 2c; see Supplementary Note 3 and Supplementary Fig. 7). As illustrated in Fig. 2a, after photoexcitation and hot-carriers transfer, electrons reside in the K valley of MoS_2_ for both K–K and K–Γ IX transitions, while holes occupy distinct valleys for these two types of IXs. Consequently, probed at the WSe_2_ band-edge transition specifically reflect the hole population of K–K IXs, and the dynamics probed at the MoS_2_ reflect the electron population of K–K and K–Γ IXs.

An additional fast decay component (<5 ps) is observed for IX electrons (Fig. 2c, indicated by the red arrow; see Supplementary Fig. 7c, d for sub-100 ps dynamics). This fast decay resembles intralayer exciton formation dynamics^35,36^ and is attributed to Coulomb interactions between hybrid holes at the Γ point and electrons at the K point of MoS_2_ (Fig. 1b). The amplitude of this fast decay decreases with increasing twist angle (~47% at 0.2° vs. 37% at 27.3°), consistent with the calculated reduction in Γ-hole distributions at larger angles (Supplementary Table 1). The longer decay lifetime of electrons compared to holes arises from the mixed decay dynamics involving momentum-indirect K–Γ transitions. Furthermore, while the hole lifetimes of K–K IXs increase significantly with twist angle due to enhanced momentum mismatch, the electron lifetimes are less affected (Fig. 2d, e; Supplementary Note 3, Supplementary Fig. 8, and Supplementary Tables 2–5). Similar asymmetric electron and hole population dynamics have been observed in WS_2_–WSe_2_ heterobilayers, associated with K–K and K–Q IXs^37^. The clear trend of increasing IX recombination lifetimes with larger twist angles, which we attribute to greater parallel momentum mismatch^33^ and expanded interlayer spacing^38^.

Figure 2e summarizes the lifetimes of interlayer electrons and holes across various pump energies above IXM threshold. In all twisted heterobilayers, excitation above the 2${E}_{{\rm{g}}\left({\rm{type}}-{\rm{II}}\right)}$ threshold produces long-lived multiple IXs with nanosecond-scale lifetimes. These lifetimes are one to two orders of magnitude longer than those of multiple intradot or intralayer excitons in previous MEG materials, such as perovskite NCs and other 2D systems^13^^,15^^,^^39–47^. This enhanced lifetime stems from the significantly reduced electron-hole wavefunction overlap in interlayer excitons compared to their intralayer counterparts, as well as from long-range (nanometer-scale) dipole-dipole interactions among them, which will be elaborated in following sections.

Figure 2f compares key performance parameters across various MEG materials, including multiple exciton lifetimes above the MEG threshold, MEG efficiency (${\eta }_{{\rm{MEG}}}$, equivalent to ${\eta }_{{\rm{IXM}}}$ for our samples), bandgap (${E}_{{\rm{g}}}$, equivalent to ${E}_{{\rm{g}}\left({\rm{type}}-{\rm{II}}\right)}$ for our samples), and the normalized threshold energy (${h\nu }_{{\rm{th}}}/{E}_{{\rm{g}}}$). The theoretical maximum ${\eta }_{{\rm{MEG}}}$ is 100%, corresponding to a step-like QY described by ${{\rm{QY}}}_{\max }=\left\lfloor \left(h\nu /{E}_{{\rm{g}}}-1\right)* {\eta }_{{\rm{MEG}}}\right\rfloor +1$ (ref. ^48^), where the floor operator (⌊⌋) denotes rounding down. A 100% ${\eta }_{{\rm{MEG}}}$ represents the ideal limit of no energy loss during multiple electron-hole pair generation. Bandgap values are included for context, as optimal values are application-dependent; for photovoltaics, MEG is most effective for bandgaps of ~0.7–1.0 eV (Supplementary Fig. 9 and Supplementary Note 4). Our MoS_2_/WSe_2_ heterobilayers uniquely combine high ${\eta }_{{\rm{IXM}}}$, a tunable type-II bandgap, a low threshold, and—most notably—exceptionally long-lived multiple interlayer excitons. This combination of properties represents a significant advance in MEG performance and establishes a new benchmark for carrier multiplication materials.

### Evidence of IXM in twisted heterobilayers

**Fig. 3:**
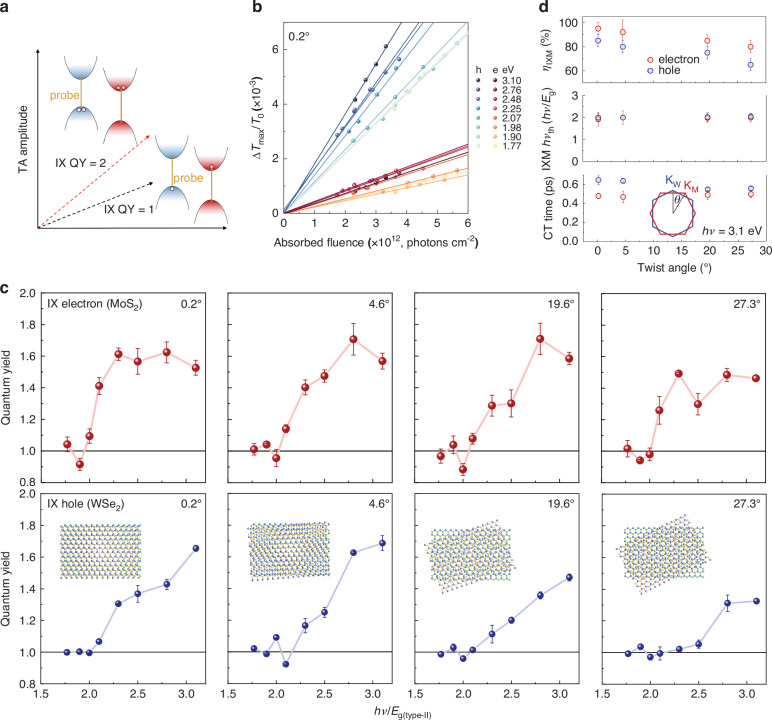
Twist-angle dependent IXM in MoS_2_/WSe_2_ heterobilayers. **a** Schematic of the IX QY determination by pump-fluence dependent TA. Probe pulses selectively monitor electrons at the MoS_2_ CBM and holes at the WSe_2_ VBM. **b** $\Delta {T}_{\max }/{T}_{0}$ as a function of absorbed pump fluence at various photon energies for the 0.2° heterobilayer. Solid lines are linear fits whose slopes correspond to the carrier QY. **c** Extracted QY for IX electrons (upper panel) and holes (lower panel) as a function of pump photon energy (normalized to ${E}_{g\left({\rm{type}}-{\rm{II}}\right)}$ of 1 eV). Error bars represent uncertainties in QY determined from the linear fits in (**b**) and Supplementary Fig. 6. **d** IXM efficiency, threshold and charge transfer (CT) time as functions of twist angle under 3.1 eV photoexcitation. Inset: Schematic representation of the twist angle in momentum space

Beyond emission multiplication, TA measurements provide further evidence and deeper insights into IXM and its twist-angle dependence (Fig. 3a). To accurately determine the interlayer exciton quantum yield (IX QY), we performed pump-fluence-dependent TA. The peak PB signals ($\Delta {T}_{\max }/{T}_{0}$) increase linearly with low excitation fluence across various photon energies (Fig. 3b; see Supplementary Figs. 6d–f and 10–17). This linearity confirms one-photon excitation without contributions from nonlinear processes. The peak PB signal is given by $\it \Delta {T}_{\max }/{T}_{{\rm{0}}}=\varPhi {\sigma }_{{\rm{PB}}}{F}_{{\rm{abs}}}$, where *Φ* is IX generation QY per absorbed photon, ${\sigma }_{{\rm{PB}}}$ is the probe absorption cross-section (constant at a fixed energy), and ${F}_{{\rm{abs}}}$ is the absorbed pump photon fluence, calibrated from microscope absorption spectra (Supplementary Fig. 4). The IX QY is thus extracted from the slope of $\Delta {T}_{\max }/{T}_{0}$ versus ${F}_{{\rm{abs}}}$ (Fig. 3a).

Analysis across multiple twist angles confirms IXM. As shown for the 0.2° sample in Fig. 3b, the slopes for both electron and hole states remain constant for $h\nu < 2{E}_{{\rm{g}}\left({\rm{type}}-{\rm{II}}\right)}$, consistent with an IX QY of 1 below the MEG threshold. Above this energy, the slopes increase, indicating enhanced IX QY. Figure 3c presents the extracted QYs as a function of $h\nu /{E}_{{\rm{g}}\left({\rm{type}}-{\rm{II}}\right)}$ for different twist angles, probed at the MoS_2_ (electrons of K–K and K–Γ IXs) and WSe_2_ (holes of K–K IXs), respectively. The QYs exceed unity at higher photon energies, confirming the generation of more than one carrier per absorbed photon in IX. The simultaneous observation of interlayer electron and hole multiplication (IEM and IHM) with a threshold near 2${E}_{{\rm{g}}\left({\rm{type}}-{\rm{II}}\right)}$, combined with the long IX lifetimes (Fig. 2e), provides definitive evidence for IXM. The extracted ${\eta }_{{\rm{IXM}}}$ values remain valid even at long delay times (e.g., 100 ps), as the subsequent decay dynamics are independent of the initial pump energy due to the long-lived nature of the IXs (Supplementary Fig. 8).

Figure 3d summarizes the averaged IEM and IHM efficiencies $\left({\bar{\eta }}_{\mathrm{IEM}/\mathrm{IHM}}=\frac{{\sum }_{i}^{n}\eta (h\nu)}{n}\right)$ for different twist angles. The IEM efficiencies are 95 ± 5%, 92 ± 10%, 85 ± 5%, and 80 ± 5%, while the IHM efficiencies are 85 ± 5%, 80 ± 5%, 75 ± 5%, and 65 ± 5% for the 0.2°, 4.6°, 19.6°, and 27.3° heterobilayers, respectively. This yields overall IXM efficiencies of ~90 ± 5%, 86 ± 8%, 80 ± 5%, and 73 ± 5%. The slightly higher ${\eta }_{{\rm{IEM}}}$ is attributed to the electron population comprising both K–K and K–Γ excitons, where the additional K–Γ IXs contribute to a higher IEM efficiency compared to IHM. Substantial momentum mismatch at larger twist angles^49^ would intuitively be expected to significantly suppress interlayer hot-carrier Coulomb scattering. The IXM process involves two key steps: (i) intralayer optical excitation of high-energy hot carriers, and (ii) interlayer scattering to generate additional electron-hole pairs, with efficient phonon scattering facilitating momentum conservation on a tens-of-femtoseconds timescale^50^. Thus, despite increased momentum mismatch at larger angles, the involvement of carrier-phonon scattering mitigates a more pronounced reduction in IXM efficiency. The QY versus $h\nu$ data further reveal that ${\eta }_{{\rm{IXM}}}$ decreases with increasing pump energy, a trend more pronounced at larger twist angles. This results from an energy-dependent competition between IXM and hot-carrier relaxation, stemming from reduced interlayer carrier-carrier scattering, potentially due to a lower density of states at higher energies (Supplementary Fig. 18 and Supplementary Note 5). A deeper understanding is provided by subsequent theoretical calculations that account for all scattering pathways.

Notably, the threshold photon energy (${h\nu }_{{\rm{th}}}$) and hot carrier transfer (HCT) appear unaffected by twist angle (Fig. 3d). The TA signal rise time under above-threshold excitation is associated with HCT (Supplementary Figs. 19, 20), which can overcome momentum mismatch even at large angles^51^. The transfer of hot electrons (holes) allows for broader exploration of *K*_*∥*_ space above the CBM (VBM), encompassing the M point where it intersects the K valley in WSe_2_ (MoS_2_) at large twist angles^52^. These energetic hot carriers then relax to the band edge, releasing their excess energy to excite additional carriers via scattering pathways that persist across all twist angles, leaving the threshold largely unaffected by momentum mismatch.

Importantly, to validate the universality of IXM across different material systems, we fabricated another type-II heterobilayer (MoS_2_/MoTe_2_) with an interlayer bandgap of 0.8 eV (Supplementary Figs. 21–23). Efficient interlayer charge separation and long-lived IX formation are similarly observed in this system. By selectively probing the CBM of MoS_2_, we quantitatively monitor the electron population dynamics of IX. The interlayer IEM QY exhibits significant jumps above 1 at the threshold energy of 1.77 eV (Supplementary Fig. 22c), confirming the presence of robust IXM in type-II heterobilayers (Supplementary Fig. 22d).

### Theoretical modeling of low-threshold IXM

To provide a theoretical foundation for the observed low-threshold IXM, we performed detailed calculations to elucidate the underlying mechanisms. Our model confirms the existence of IX multiplication channels with a threshold energy of $2{E}_{{\rm{g}}\left({\rm{type}}-{\rm{II}}\right)}$ across various twisted heterobilayers, mediated by interlayer carrier-carrier scattering. The band structure of the heterobilayer is described using an ab-initio-based tight-binding model^53^, with band offsets adjusted to align with experimental observations.

**Fig. 4:**
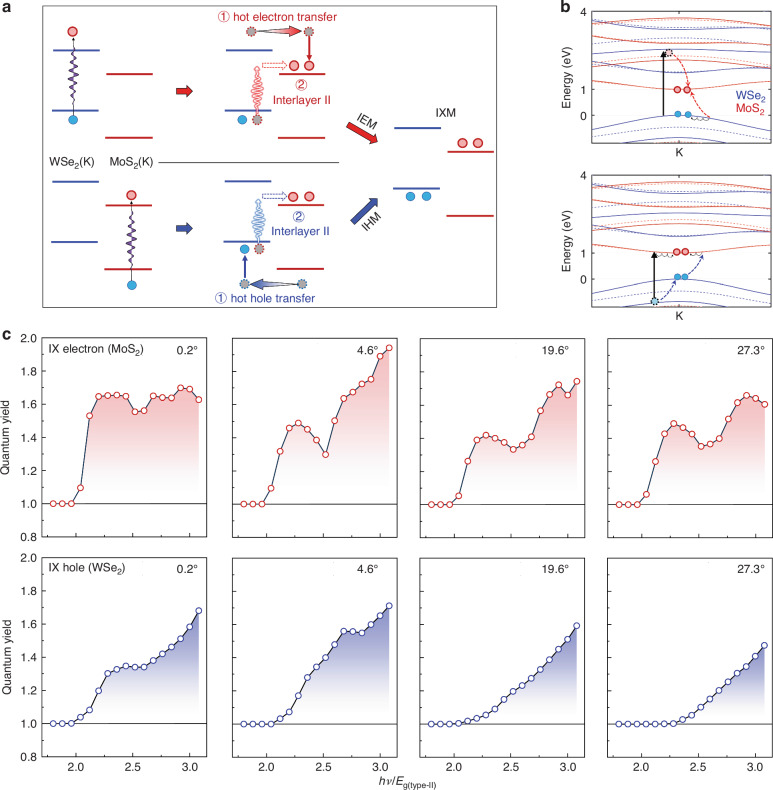
Theorical modeling of IXM in twisted MoS_2_/WSe_2_ heterobilayers. **a** Schematic of the IXM mechanism via interlayer hot-electron scattering (top) and hot-hole scattering (bottom). Red and blue circles represent electrons and holes, respectively; II denotes impact ionization. **b** Calculated band structure of the 0.2° heterobilayer showing a direct fundamental gap. Solid and dashed lines represent spin-up and spin-down bands, respectively. Dashed arrows indicate hot-carrier relaxation to the band extremum, enabling secondary carrier generation across the type-II gap via impact ionization. **c** Calculated interlayer electron multiplication (IEM) quantum yield (upper panel) and interlayer hole multiplication (IHM) quantum yield (lower panel) as a function of normalized photon energy for different twist angles

The IXM process can be classified into hot-electron-induced IEM and hot-hole-induced IHM, each involving two primary steps (Fig. 4a). In the IEM process: (1) A valence electron in WSe_2_ is excited to a high-energy state within its conduction band. (2) This hot electron transfers to the MoS_2_ layer. If its excess energy exceeds ${E}_{{\rm{g}}\left({\rm{type}}-{\rm{II}}\right)}$, it can undergo impact scattering, exciting a second valence electron from WSe_2_ to the CBM of MoS_2_ and thereby generating an additional electron-hole pair via impact ionization. A analogous process occurs for IHM (Fig. 4a, lower panel). Figure 4b displays the calculated band structure of the 0.2° heterobilayer, illustrating how IXM can occur as electrons scatter from the K/K’ valley of WSe_2_ to the K/K’ valley of MoS_2_, conserving both energy and momentum.

Within this framework, the QY for the interlayer multiplication process is calculated by^54^

1 $${\rm{QY}}\left(h\nu \right)=\frac{1}{{N}_{k}^{2}}\mathop{\sum }\limits_{X}{D}_{X}\left(h\nu \right)\frac{2{W}_{X}+{\gamma }_{{\rm{cool}}}}{{{{W}}}_{X}+{\gamma }_{{\rm{cool}}}}$$

where ${N}_{k}^{2}={10}^{4}$ is the number of $k$-point samplings in the 2D Brillouin zone, and ${\gamma }_{{\rm{cool}}}$ is the intralayer hot-carrier cooling rate. For a transition $X=\left({W;i},{m;}{\bf{k}}\right)$, the term ${D}_{X}\left(h\nu \right)={\left|{d}_{{\bf{k}}}^{{im}}\right|}^{2}\delta \left({E}_{m{{\bf{k}}}}-{E}_{i{{\bf{k}}}}-h\nu \right)$ denotes the absorption rate for an electron excited from the initial state |*W*,*i*,**k**〉 to the final state |*W*,*m*,**k**〉 by a photon of energy *hν*. The term ${W}_{X}$ represents the scattering rate between two hot carriers, given by:

2 $${W}_{X}={\sum }_{{m}^{{\prime} }n{n}^{{\prime} }}{\sum }_{{{\bf{k}}}^{{\prime} }{\bf{q}}{{\bf{q}}}^{{\prime} }}{\sum }_{{\bf{G}}}V\left({{\bf{k}}}^{{\prime} }-{\bf{k}}-{\bf{G}}\right){I}_{m{\bf{k}},n{\bf{q}}}^{{m}^{{\prime} }{{\bf{k}}}^{{\prime} },{n}^{{\prime} }{{\bf{q}}}^{{\prime} }}{\delta }_{{\bf{k}}+{\bf{q}}+{\bf{G}}}^{{{\bf{k}}}^{{\prime} }+{{\bf{q}}}^{{\prime} }}\delta \left({E}_{m{\bf{k}}}+{E}_{n{\bf{q}}}-{E}_{{m}^{{\prime} }{{\bf{k}}}^{{\prime} }}-{E}_{{n}^{{\prime} }{{\bf{q}}}^{{\prime} }}\right)$$

This formulation includes all scattering pathways that ensure energy and momentum conservation in twisted heterobilayers. Here, **G** is a reciprocal lattice vector. The screened Coulomb interaction in *k* space is:

3 $$V\left({\bf{k}}\right)=\frac{{e}^{2}}{2{\epsilon }_{0}\left|{\bf{k}}\right|}{\epsilon }_{{\rm{eff}}}^{-1}\left({\bf{k}}\right)$$

where ${\epsilon }_{0}$ is the vacuum permittivity and ${\epsilon }_{{\rm{eff}}}$ is the effective dielectric function obtained by solving Poisson’s equation^55^. The interaction strength decreases with increasing momentum difference |**k**|, which accounts for the reduced IXM efficiency at larger twist angles. The overlap matrix elements ${I}_{m{\bf{k}},n{\bf{q}}}^{{m}^{{\prime} }{{\bf{k}}}^{{\prime} },{n}^{{\prime} }{{\bf{q}}}^{{\prime} }}$ are approximated as a constant ${I}_{0}\left(\theta \right)$ for a given twist angle *θ*. In our simulations, the ratio ${I}_{0}\left(\theta \right)/{\gamma }_{{\rm{cool}}}$ in Eq. (1) is adjusted to account for variations in the cooling rate and interlayer wavefunction overlap at different twist angles. We also define the density of trion states (DOTS) as:

4 $${\rho }_{T}=\mathop{\sum }\limits_{X}\mathop{\sum }\limits_{{m}^{{\prime} }n{n}^{{\prime} }}\mathop{\sum }\limits_{{{\bf{k}}}^{{\prime} }{{\bf{qq}}}^{{\prime} }}\mathop{\sum }\limits_{{\bf{G}}}{\delta }_{{\bf{k}}+{\bf{q}}+{\bf{G}}}^{{{\bf{k}}}^{{\prime} }+{{\bf{q}}}^{{\prime} }}\delta \left({E}_{m{\bf{k}}}+{E}_{n{\bf{q}}}-{E}_{{m}^{{\prime} }{{\bf{k}}}^{{\prime} }}-{E}_{{n}^{{\prime} }{{\bf{q}}}^{{\prime} }}\right)$$

to characterize the number of scattering pathways.

The results in Fig. 4c clearly demonstrate both IEM and IHM when $h\nu \ge 2{E}_{{\rm{g}}\left({\rm{type}}-{\rm{II}}\right)}$, aligning well with experimental measurements. The consistent low threshold indicates that energy conservation is achieved by leveraging hot-carrier excess energies from band offset energies. Additionally, twist-angle-induced momentum mismatches have an unexpected small effect on the threshold of IXM, indicating the strong hot-carrier interlayer transfer and intervalley phonon-assisted impact ionization^56^. The higher IXM QY at small twist angles results from better interlayer alignment in *k* space, resulting from the stronger Coulomb scattering. The characteristic peaks in IEM (Figs. 3c and 4c upper panels) and the subtle peaks in IHM (Figs. 3c and 4c lower panels) reflect the features of DOTS as a function of photon energy (Supplementary Fig. 24).

### Long-range attractive interaction of multiple interlayer excitons

**Fig. 5:**
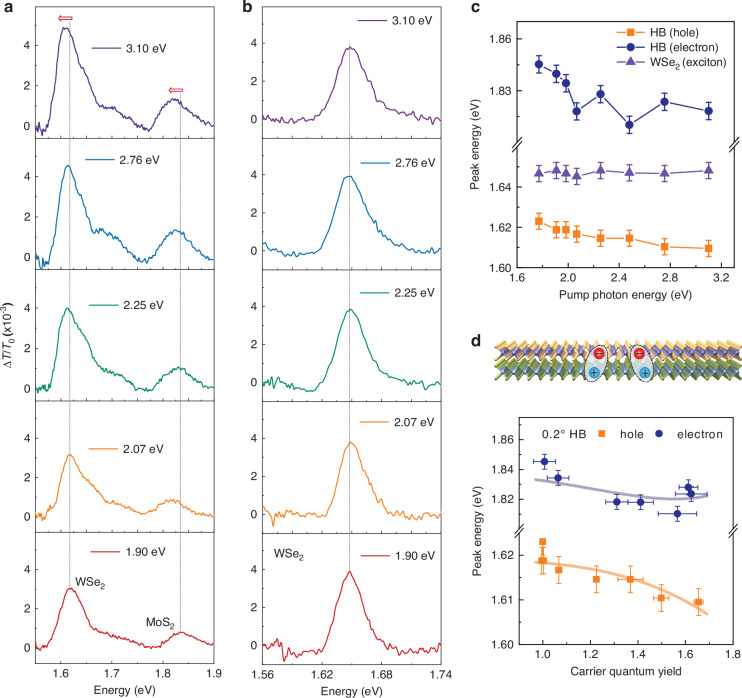
Long-range attractive interactions between multiple IXs. **a** TA spectra of the 0.2° MoS_2_/WSe_2_ heterobilayer (HB) at a 1.5 ps delay, measured at a constant absorbed photon density of 3.0 × 10^12 ^cm^−2^. Dashed lines indicate PB peak positions. **b** Corresponding TA spectra of monolayer WSe_2_ under identical conditions for comparison. **c** Transition energies for IX electrons and holes as functions of pump photon energy, and **d** IX QY with theoretical fits (solid curves; see main text). Error bars represent uncertainties in transition energy (vertical) and QY (horizontal). Inset: Schematic of two IXs exhibiting long-range dipole-dipole interactions

Beyond the quantitative correlation between TA amplitude and IX density, distinct spectral features provide deeper insights into IXM and the interactions among the generated multiple IXs. Figure 5a reveals clear signatures of enhanced IX populations and interactions. The PB amplitudes probed at the MoS_2_ CBM and WSe_2_ VBM for excitation above the ${h\nu }_{{\rm{th}}}$ threshold are significantly higher than those below it, confirming an increased IX population for a fixed absorbed photon density. Furthermore, the electron and hole states above the threshold exhibit a pronounced redshift (Fig. 5a), a phenomenon absent in individual WSe_2_ monolayers across the same pump energy range (Fig. 5b). We can rule out laser-induced thermal effects or hot-carrier dynamics as the origin of this shift. This is evidenced by the stable position of the WSe_2_ intralayer exciton resonance, which shows no measurable energy shift under identical pump photon energies (Fig. 5b) and at high pump fluence (Supplementary Fig. 25).

Figure 5c summarizes the energy positions of the IX PB peaks for electrons and holes in the 0.2° heterobilayer, compared to the monolayer WSe_2_ peak. This redshift is only observed under high pump fluence (>20 × 10^12^ photons cm^−2^) when exciting below the IXM threshold at 1.9 eV, whereas it appears even at low fluence for above-threshold excitation (Supplementary Fig. 26 and Supplementary Note 6). This fluence dependence further validates that IXM occurs efficiently under low fluence once the photon energy threshold is exceeded. We attribute the redshift in the heterobilayer to attractive interactions arising from quantum-exchange correlation effects^57^ between the single-photon-generated multiple IXs. We define the redshift of the transition energy under above-threshold excitation as Δ*E*(*hν*) = *E*(2*E*_g_)−*E*(*hν*). The experimental data (Fig. 5d, dots and squares) are well-described by a Lennard-Jones potential model (solid curve)^57,58^:

5 $$\Delta E\left(h\nu \right)=\varepsilon \left\{{\left[{r}_{s}/r\right]}^{m}-{\left[{r}_{s}/r\right]}^{k}\right\},r={\left[{n}_{0}\pi {\rm{QY}}\left(h\nu \right)\right]}^{-\frac{1}{2}}$$

Here, the first term represents short-range Pauli repulsion and the second term long-range vdW attraction (Fig. 5d, solid lines). The variable *r* is the average distance between electrons or holes in the multiple IX ensemble, related to the charge carrier density $n\left(h\nu \right)\pi {r}^{2}=1$. Given that $n\left(h\nu \right)\propto {n}_{0}\cdot {\rm{QY}}\left(h\nu \right)$ due to IXM, where *n*_0_ is the initial concentration of photoexcited charge carriers (on the order of 10^12^cm^-2^), the distance *r* decreases as the QY increases. The parameter *r*_s_ (s *=* h or e) represents the effective Bohr radius of the exciton.

This redshift is also observed in heterobilayers with other twist angles (Supplementary Figs. 27, 28 and Supplementary Table 6). From the fits, we determine an interaction radius *r*_h_ of ~6 nm for holes and *r*_e_ of ~4 nm for electrons in the 0.2° heterobilayer. These large distances, significantly exceeding the heterobilayer thickness, signify long-range attractive dipole-dipole interactions between the stable multiple IXs^57^. Furthermore, *r*_h_ reaches a minimum at 4.6° before increasing, while *r*_e_ rises slightly with twist angle (Supplementary Table 6). Correlating the behavior of *r*_s_ with the carrier multiplication QYs from Fig. 3c reveals a general trend: higher carrier multiplication QY correspond to shorter interlayer attractive interaction distances, enhancing the attractive interactions.

### IXM-induced photocurrent multiplication

**Fig. 6:**
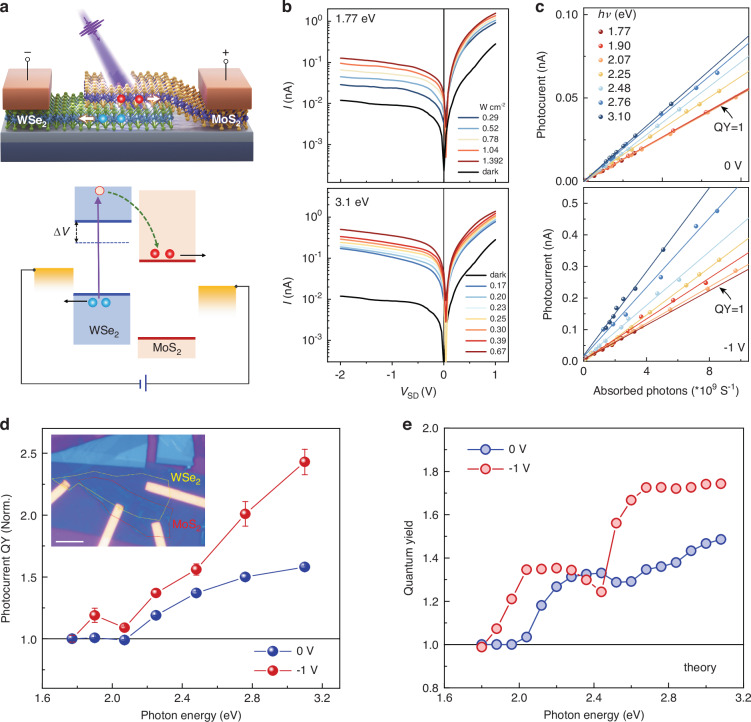
Photocurrent multiplication in a MoS_2_/WSe_2_ heterobilayer photodiode. **a** Device schematic (top) and corresponding energy band diagram under applied bias, illustrating the interlayer exciton multiplication (IXM) process (bottom). **b** Current-voltage characteristics under 1.77 eV (top) and 3.1 eV (bottom) illumination at different power intensities. Dark current is shown for reference. **c** Photocurrent versus absorbed photon flux at various excitation energies for applied biases of 0 V (top) and -1 V (bottom). **d** Experimental photocurrent quantum yield as a function of pump photon energy at 0 V and −1 V. Inset: optical micrograph of the 3°-twisted heterobilayer device. Scale bar: 5 μm. Error bars indicate uncertainties in QY determination. **e** Theoretical quantum yield versus pump photon energy simulated for 0 V and −1 V biases

Having established that multiple IXs generated by high-energy photons exhibit long lifetimes—a critical property for efficient carrier extraction—we next demonstrate photocurrent multiplication induced by IXM in a functional device. We fabricated a 3°-twisted MoS_2_/WSe_2_ photodiode and evaluated its photocurrent QY under varying photon energies (Fig. 6a). The application of a voltage to the WSe_2_ layer creates a lateral electric field that separates and collects the electron-hole pairs generated in the heterobilayer. Notably, under a negative bias, photoexcited hot electrons in the WSe_2_ conduction band gain additional energy ($\Delta V$) due to the shift of the conduction band, effectively lowering the energy required for impact ionization (Fig. 6a, lower panel). Current-voltage (*I-V*) characterization shows typical diode behavior in the dark and a clear photovoltaic response under illumination, with an open-circuit voltage in the millivolt range (Fig. 6b; Supplementary Figs. 29, 30). The photocurrent increases linearly with absorbed photon flux across all excitation energies (Fig. 6c), confirming that the response is governed by single-photon processes. The slope of these curves corresponds directly to the photocurrent QY at each photon energy. The internal quantum efficiency (IQE) nearly doubles, increasing to ~42% at *V* = −1 V as the photon energy rises from 2 eV to 3.1 eV (Supplementary Fig. 31a).

Figure 6d displays the normalized internal photocurrent QY at *V* = 0 V (self-powered mode), revealing a clear threshold behavior where the QY exceeds unity for photon energies above 2${E}_{g\left({\rm{type}}-{\rm{II}}\right)}$). As the electric field is constant for these measurements, any field-induced effects on exciton dynamics are consistent and accounted for in the normalization. This result directly correlates with the optical measurements in Fig. 3, confirming that the observed IXM translates into a measurable photocurrent gain. Applying a negative bias of −1 V further enhances the effect, yielding both a lower experimental IXM threshold of 1.8 eV and a higher photocurrent QY (Fig. 6d). This is attributed to the additional potential energy imparted to the carriers, which reduces the net energy required for impact ionization. Our theoretical calculations corroborate this, showing enhanced IXM efficiency and a lower threshold under negative bias on WSe_2_ (Fig. 6e). At a given pump energy, carriers under negative bias possess greater excess energy, opening more scattering pathways and increasing the QY. This IXM-driven enhancement directly improves device performance. Due to the multiplication process, the external quantum efficiency (EQE) and responsivity increase from 0.9% and 4.9 mA W^−1^ at 2 eV to 4.7% and 15.4 mA W^−1^ at 3.1 eV under a −1 V bias (Supplementary Fig. 31b, c).

## Discussion

In summary, we have demonstrated highly efficient, low-threshold IXM in twisted type-II TMDC heterobilayers. The IXM process features a threshold as low as twice the interlayer bandgap, approaching the fundamental energy conservation limit. Our combined experimental and theoretical work reveals that this low threshold is robust against twist-angle-induced momentum mismatch, enabled by efficient hot-carrier interlayer transfer and phonon-assisted scattering. We achieved a peak IXM efficiency of ~90% in a 0.2° heterobilayer, where shorter inter-carrier distances enhance attractive interactions. This efficiency gradually decreases with larger twist angles and higher photon energies due to reduced interlayer Coulomb scattering. Furthermore, we successfully translated this optical multiplication into a functional device, demonstrating IXM-induced photocurrent gain. The photocurrent QY exhibits a clear multiplication threshold at $2{E}_{{\rm{g}}\left({\rm{type}}-{\rm{II}}\right)}$ in a self-powered photodiode, which can be further reduced to 1.8${E}_{{\rm{g}}\left({\rm{type}}-{\rm{II}}\right)}$ under a modest reverse bias. Our findings establish a new paradigm for carrier multiplication and pave the way for developing highly efficient optoelectronic devices, such as photodetectors and solar cells, that surpass conventional efficiency limits. Additionally, the observed long-range interactions between stable multiple IXs highlight the potential of these systems for exploring novel quantum optical phenomena.

## Materials and methods

### Sample fabrication

Monolayers of MoS_2_, WSe_2_, and MoTe_2_, along with few-layer h-BN flakes, were mechanically exfoliated from their bulk crystals using adhesive tape. The exfoliated flakes were transferred onto a polydimethylsiloxane (PDMS) stamp, and their thicknesses were identified based on optical contrast under an optical microscope. Twisted MoS_2_/WSe_2_ and MoS_2_/MoTe_2_ heterobilayers were fabricated using a precision dry-transfer technique under optical microscopy guidance. The heterostructures were fully encapsulated by top and bottom h-BN layers. Specifically, a selected h-BN flake was first transferred onto a quartz substrate. Subsequently, MoS_2_, WSe_2_ (or MoTe_2_), and another h-BN flake were sequentially transferred with meticulous alignment to achieve the target twist angles. Following each transfer step, the sample was annealed in a vacuum at 473 K for 3 h to enhance interfacial cleanliness and contact quality.

### Device fabrication and characterization

Monolayer flakes of MoS_2_, WSe_2_, and MoTe_2_ were mechanically exfoliated onto PDMS stamps. Selected flakes were then precisely aligned and vertically stacked using a dry-transfer method onto a Si substrate with a 300 nm thermal oxide layer. The substrate was pre-cleaned by sequential ultrasonication in acetone, isopropyl alcohol, and deionized water. The heterostructure was subsequently annealed at 200 °C for 2 h in vacuum to improve interfacial contact. Cr/Au electrodes (10/50 nm) were then fabricated on the MoS_2_/WSe_2_ or MoS_2_/MoTe_2_ heterobilayers via standard electron-beam lithography, thermal evaporation, and a lift-off process. Finally, a few-layer h-BN flake was transferred onto the active channel as a top-gate dielectric, followed by a second annealing step at 200 °C for 2 h. Electrical characterization of the devices was performed using a Keithley B1500A semiconductor parameter analyzer in a Lake Shore cryogenic probe station under a vacuum of 10^-6^ Torr. Measurements were conducted in both dark and illuminated conditions. The illumination source was a fiber-coupled laser system, and the laser power was calibrated before each measurement using a Newport 843-R optical power meter with a PD300-UV photodetector. All electrical measurements were performed at room temperature.

### Angle-resolved second-harmonic-generation

The SHG measurements were conducted using a home-built inverted microscopy system. A linearly polarized femtosecond laser light with the following parameters: wavelength of 800 nm, repetition rate of 80 MHz, pulse duration of 100 fs, and power of 1 mW, was focused onto the monolayer region of either MoS_2_ or WSe_2_ using a 50× objective. The reflected 400 nm light was collected by the same objective and directed through a linear polarizer and a 400 nm narrow band filter before reaching a Spectra Pro 2300i Acton equipped with a CCD camera (Pixis 256BR, Princeton Instruments). During the measurements, the polarization direction of both the excitation light and the collected light was fixed, while the heterobilayer sample was rotated. The absolute rotational angle is given in arbitrary units. The angular-dependent SHG intensity (I) was fitted using the equation *I* = *I*_max_ cos^2^[3(θ + φ)], where φ is between 0° and 30°. Since SHG intensity measurements are not sensitive to phase, they produce a 6-fold symmetry pattern. The extracted twist angle Δ*ϕ* = |*φ*_W_ – φ_M_| for all heterobilayers ranged between 0° and 30°. It is important to note that all monolayer samples exhibited a near-perfect 6-fold symmetry, which confirms their single crystal nature. All optical measurements were performed at room temperature.

### Steady-state absorption microscopy

The absorption spectra of the monolayers and heterobilayers were measured using a laser-driven broadband light source (EQ-77-fc, Energetiq). The light was focused to a spot size of ~2 µm at the sample plane. The transmitted light was collected by a 50× microscope objective (NA = 0.5) and directed to a spectrometer (Spectra Pro 2300i, Acton) coupled with a CCD camera (Pixis 256BR, Princeton Instruments). Absorbance (*A*) was calculated using the formula *A* = log_10_(*I*_0_/*I*), where *I* is the transmitted intensity through the sample (TMD flake and substrate) and *I*_0_ is the reference intensity transmitted through the bare sapphire substrate.

### Femtosecond transient absorption microscopy

Carrier dynamics were investigated using a femtosecond TA microscope in a transmission geometry. The system was driven by a 1 kHz Ti:sapphire amplified laser system, producing 50 fs pulses centered at 800 nm. The output was split into two paths: the majority of the pulse energy pumped an optical parametric amplifier to generate a wavelength-tunable pump beam, which was mechanically chopped at 500 Hz. The remaining 800 nm fundamental beam was focused onto a sapphire crystal to generate a white-light continuum, serving as the broadband probe. The pump and probe pulses were spatially combined using a beam splitter and temporally and spatially overlapped at the sample position using a microscope objective. The time delay between them was controlled by a motorized linear stage with a retroreflector in the probe path. After transmitting through the sample, the probe beam was dispersed by a spectrometer and detected by a CMOS sensor, covering the visible spectrum (400–800 nm). This configuration allowed for the measurement of the differential transmittance (Δ*T*/*T*_0_), which reflects pump-induced changes in the sample’s complex refractive index and correlates with the photocarrier density. All measurements were conducted at room temperature under ambient conditions.

### First-principles calculations

First-principles calculations based on density functional theory (DFT) were performed using the *Quantum ESPRESSO* package to investigate the electronic structure of twisted MoS_2_/WSe_2_ heterobilayers. Commensurate supercells for various twist angles were generated using the *Twister* tool^59^. A vacuum spacing of 25 Å was introduced along the out-of-plane direction to eliminate spurious interactions between periodic images. The Perdew–Burke–Ernzerhof exchange-correlation functional within the generalized gradient approximation was employed^60^. Norm-conserving pseudopotentials were used for Mo and W atoms, while ultrasoft pseudopotentials were used for S and Se atoms. The kinetic energy cutoffs were set to 40 Ry for the wavefunctions and 200 Ry for the charge density. For each supercell, self-consistent field calculations were performed using a 9 × 9× 1 Monkhorst-Pack k-point mesh, with a Gaussian smearing of 0.01 Ry. The total energy was converged to within 10^−8 ^Ry. To analyze the layer hybridization, the partial charge densities of states at high-symmetry k-points (e.g., K and Γ) were computed. The spatial distribution of these states across the MoS_2_ and WSe_2_ layers was quantified by integrating the partial charge density within each monolayer’s region^31^. The boundary between the two layers was defined by the minimum in the plane-averaged charge density along the out-of-plane direction.

## Supplementary information

Supplementary Information

## Data Availability

The data that support the findings of this study are available from the corresponding author upon reasonable request.

## References

[1] Zhang, Y. Z. et al. Internal quantum efficiency higher than 100% achieved by combining doping and quantum effects for photocatalytic overall water splitting. *Nat. Energy* **8**, 504–514 (2023).

[2] Cirloganu, C. M. et al. Enhanced carrier multiplication in engineered quasi-type-II quantum dots. *Nat. Commun.* **5**, 4148 (2014). 24938462 10.1038/ncomms5148 PMC4083434

[3] Arnold, D., Cartier, E. & Dimaria, D. J. Acoustic-phonon runaway and impact ionization by hot electrons in silicon dioxide. *Phys. Rev. B* **45**, 1477–1480 (1992). 10.1103/physrevb.45.1477 10001636

[4] Cao, L. N. et al. Experimental characterization of impact ionization coefficients for electrons and holes in GaN grown on bulk GaN substrates. *Appl. Phys. Lett.* **112**, 262103 (2018).

[5] Wolf, M. et al. Solar cell efficiency and carrier multiplication in Si_1-x_Ge_*x*_ alloys. *J. Appl. Phys.* **83**, 4213–4221 (1998).

[6] Christensen, O. Quantum efficiency of the internal photoelectric effect in silicon and germanium. *J. Appl. Phys.* **47**, 689–695 (1976).

[7] Robbins, D. J. Aspects of the theory of impact ionization in semiconductors (I). *Phys. Status Solidi (B)* **97**, 9–50 (1980).

[8] Schaller, R. D. & Klimov, V. I. High efficiency carrier multiplication in PbSe nanocrystals: implications for solar energy conversion. *Phys. Rev. Lett.* **92**, 186601 (2004). 15169518 10.1103/PhysRevLett.92.186601

[9] Midgett, A. G. et al. Size and composition dependent multiple exciton generation efficiency in PbS, PbSe, and PbS_*x*_Se_1-*x*_ alloyed quantum dots. *Nano Lett.* **13**, 3078–3085 (2013). 23750998 10.1021/nl4009748

[10] Beard, M. C. et al. Third generation photovoltaics based on multiple exciton generation in quantum confined semiconductors. *Acc. Chem. Res.* **46**, 1252–1260 (2013). 23113604 10.1021/ar3001958

[11] Nozik, A. J. et al. Semiconductor quantum dots and quantum dot arrays and applications of multiple exciton generation to third-generation photovoltaic solar cells. *Chem. Rev.* **110**, 6873–6890 (2010). 20945911 10.1021/cr900289f

[12] Trinh, M. T. et al. Direct generation of multiple excitons in adjacent silicon nanocrystals revealed by induced absorption. *Nat. Photonics* **6**, 316–321 (2012).

[13] Saeed, S. et al. Carrier multiplication in germanium nanocrystals. *Light Sci. Appl.* **4**, e251 (2015).

[14] Zheng, W. H., Bonn, M. & Wang, H. I. Photoconductivity multiplication in semiconducting few-layer MoTe_2_. *Nano Lett.* **20**, 5807–5813 (2020). 32697101 10.1021/acs.nanolett.0c01693 PMC7458477

[15] Kim, J. H. et al. Carrier multiplication in van der Waals layered transition metal dichalcogenides. *Nat. Commun.* **10**, 5488 (2019). 31792222 10.1038/s41467-019-13325-9 PMC6889496

[16] Chen, Y. F. et al. Multiple exciton generation in tin–lead halide perovskite nanocrystals for photocurrent quantum efficiency enhancement. *Nat. Photonics* **16**, 485–490 (2022).

[17] Schaibley, J. R. et al. Valleytronics in 2D materials. *Nat. Rev. Mater.* **1**, 16055 (2016).

[18] Sierra, J. F. et al. Van der Waals heterostructures for spintronics and opto-spintronics. *Nat. Nanotechnol.* **16**, 856–868 (2021). 34282312 10.1038/s41565-021-00936-x

[19] Liu, X. L. & Hersam, M. C. 2D materials for quantum information science. *Nat. Rev. Mater.* **4**, 669–684 (2019).

[20] Jiang, Y. et al. Interlayer exciton formation, relaxation, and transport in TMD van der Waals heterostructures. *Light Sci. Appl.* **10**, 72 (2021). 33811214 10.1038/s41377-021-00500-1 PMC8018964

[21] Rivera, P. et al. Interlayer valley excitons in heterobilayers of transition metal dichalcogenides. *Nat. Nanotechnol.* **13**, 1004–1015 (2018). 30104622 10.1038/s41565-018-0193-0

[22] Bian, A. et al. Dynamics of charge-transfer excitons in a transition metal dichalcogenide heterostructure. *Nanoscale* **12**, 8485–8492 (2020). 32242201 10.1039/d0nr01924k

[23] Ceballos, F. et al. Ultrafast charge separation and indirect exciton formation in a MoS_2_–MoSe_2_ van der Waals heterostructure. *ACS Nano* **8**, 12717–12724 (2014). 25402669 10.1021/nn505736z

[24] Ceballos, F. et al. Probing charge transfer excitons in a MoSe_2_–WS_2_ van der Waals heterostructure. *Nanoscale* **7**, 17523–17528 (2015). 26444979 10.1039/c5nr04723d

[25] Jauregui, L. A. et al. Electrical control of interlayer exciton dynamics in atomically thin heterostructures. *Science* **366**, 870–875 (2019). 31727834 10.1126/science.aaw4194

[26] Joe, A. Y. et al. Controlled interlayer exciton ionization in an electrostatic trap in atomically thin heterostructures. *Nat. Commun.* **15**, 6743 (2024). 39112505 10.1038/s41467-024-51128-9 PMC11306233

[27] Kumar, N. et al. Second harmonic microscopy of monolayer MoS_2_. *Phys. Rev. B* **87**, 161403 (2013).

[28] Malard, L. M. et al. Observation of intense second harmonic generation from MoS_2_ atomic crystals. *Phys. Rev. B* **87**, 201401 (2013).

[29] Li, Y. L. et al. Probing symmetry properties of few-layer MoS_2_ and h-BN by optical second-harmonic generation. *Nano Lett.* **13**, 3329–3333 (2013). 23718906 10.1021/nl401561r

[30] Karni, O. et al. Infrared interlayer exciton emission in MoS_2_/WSe_2_ heterostructures. *Phys. Rev. Lett.* **123**, 247402 (2019). 31922842 10.1103/PhysRevLett.123.247402

[31] Kunstmann, J. et al. Momentum-space indirect interlayer excitons in transition-metal dichalcogenide van der Waals heterostructures. *Nat. Phys.* **14**, 801–805 (2018).

[32] Chiu, M. H. et al. Determination of band alignment in the single-layer MoS_2_/WSe_2_ heterojunction. *Nat. Commun.* **6**, 7666 (2015). 26179885 10.1038/ncomms8666 PMC4518320

[33] Choi, J. et al. Twist angle-dependent interlayer exciton lifetimes in van der Waals heterostructures. *Phys. Rev. Lett.* **126**, 047401 (2021). 33576642 10.1103/PhysRevLett.126.047401

[34] Hong, X. P. et al. Ultrafast charge transfer in atomically thin MoS_2_/WS_2_ heterostructures. *Nat. Nanotechnol.* **9**, 682–686 (2014). 25150718 10.1038/nnano.2014.167

[35] Ceballos, F. et al. Exciton formation in monolayer transition metal dichalcogenides. *Nanoscale* **8**, 11681–11688 (2016). 27219022 10.1039/c6nr02516a

[36] Steinleitner, P. et al. Direct observation of ultrafast exciton formation in a monolayer of WSe_2_. *Nano Lett.* **17**, 1455–1460 (2017). 28182430 10.1021/acs.nanolett.6b04422

[37] Yuan, L. et al. Twist-angle-dependent interlayer exciton diffusion in WS_2_–WSe_2_ heterobilayers. *Nat. Mater.* **19**, 617–623 (2020). 32393806 10.1038/s41563-020-0670-3

[38] Yuan, Y. J. et al. Probing the twist-controlled interlayer coupling in artificially stacked transition metal dichalcogenide bilayers by second-harmonic generation. *ACS Nano* **17**, 17897–17907 (2023). 37698446 10.1021/acsnano.3c03795

[39] Li, Y. L. et al. Biexciton Auger recombination in mono-dispersed, quantum-confined CsPbBr_3_ perovskite nanocrystals obeys universal volume-scaling. *Nano Res.* **12**, 619–623 (2019).

[40] Meng, J. et al. Tailoring auger recombination dynamics in CsPbI_3_ perovskite nanocrystals via transition metal doping. *Nano Lett.* **24**, 8386–8393 (2024). 38934731 10.1021/acs.nanolett.4c02032

[41] Li, M. J. et al. Low threshold and efficient multiple exciton generation in halide perovskite nanocrystals. *Nat. Commun.* **9**, 4197 (2018). 30305633 10.1038/s41467-018-06596-1 PMC6180109

[42] Christodoulou, S. et al. Single-exciton gain and stimulated emission across the infrared telecom band from robust heavily doped PbS colloidal quantum dots. *Nano Lett.* **20**, 5909–5915 (2020). 32662655 10.1021/acs.nanolett.0c01859

[43] Yang, W. X. et al. Surface passivation extends single and biexciton lifetimes of InP quantum dots. *Chem. Sci.* **11**, 5779–5789 (2020). 32832054 10.1039/d0sc01039a PMC7416692

[44] Zhou, Q. H. et al. Highly efficient multiple exciton generation and harvesting in few-layer black phosphorus and heterostructure. *Nano Lett.* **20**, 8212–8219 (2020). 33044075 10.1021/acs.nanolett.0c03328

[45] Al-Otaify, A. et al. Multiple exciton generation and ultrafast exciton dynamics in HgTe colloidal quantum dots. *Phys. Chem. Chem. Phys.* **15**, 16864–16873 (2013). 23999734 10.1039/c3cp52574k

[46] Ghosh, G. et al. Carrier multiplication and photoexcited many-body states in solution-processed 2H-MoSe_2_. *ACS Nano* **19**, 10347–10358 (2025). 40047396 10.1021/acsnano.4c18254 PMC11924332

[47] Karmakar, R. et al. Multiple carrier generation at an exceptionally low energy threshold. *Phys. Rev. Lett.* **134**, 026903 (2025). 39913820 10.1103/PhysRevLett.134.026903

[48] Beard, M. C. et al. Comparing multiple exciton generation in quantum dots to impact ionization in bulk semiconductors: implications for enhancement of solar energy conversion. *Nano Lett.* **10**, 3019–3027 (2010). 20698615 10.1021/nl101490z

[49] Zhu, Y. H. et al. The twist angle has weak influence on charge separation and strong influence on recombination in the MoS_2_/WS_2_ bilayer: ab initio quantum dynamics. *J. Mater. Chem. A* **10**, 8324–8333 (2022).

[50] Liu, F., Li, Q. Y. & Zhu, X. Y. Direct determination of momentum-resolved electron transfer in the photoexcited van der Waals heterobilayer WS_2_/MoS_2_. *Phys. Rev. B* **101**, 201405 (2020).

[51] Ji, Z. H. et al. Robust stacking-independent ultrafast charge transfer in MoS_2_/WS_2_ bilayers. *ACS Nano* **11**, 12020–12026 (2017). 29116758 10.1021/acsnano.7b04541

[52] Zhu, H. M. et al. Interfacial charge transfer circumventing momentum mismatch at two-dimensional van der Waals heterojunctions. *Nano Lett.* **17**, 3591–3598 (2017). 28481550 10.1021/acs.nanolett.7b00748

[53] Fang, S. A. et al. Electronic structure theory of strained two-dimensional materials with hexagonal symmetry. *Phys. Rev. B* **98**, 075106 (2018).

[54] Isler, M. Phonon-assisted impact ionization of electrons in In_0.53_Ga_0.47_As. *Phys. Rev. B* **63**, 115209 (2001).

[55] Eshet, H. et al. Theory of highly efficient multiexciton generation in type-II nanorods. *Nat. Commun.* **7**, 13178 (2016). 27725668 10.1038/ncomms13178 PMC5062596

[56] Florian, M. et al. The dielectric impact of layer distances on exciton and trion binding energies in van der Waals heterostructures. *Nano Lett.* **18**, 2725–2732 (2018). 29558797 10.1021/acs.nanolett.8b00840

[57] Sun, X. Q. et al. Enhanced interactions of interlayer excitons in free-standing heterobilayers. *Nature* **610**, 478–484 (2022). 36224395 10.1038/s41586-022-05193-z

[58] Sie, E. J. et al. Observation of exciton redshift–blueshift crossover in monolayer WS_2_. *Nano Lett.* **17**, 4210–4216 (2017). 28621953 10.1021/acs.nanolett.7b01034

[59] Naik, S. et al. Twister: construction and structural relaxation of commensurate moiré superlattices. *Comput. Phys. Commun.* **271**, 108184 (2022).

[60] Perdew, J. P., Burke, K. & Ernzerhof, M. Generalized gradient approximation made simple. *Phys. Rev. Lett.* **77**, 3865–3868 (1996). 10062328 10.1103/PhysRevLett.77.3865

