# Supplementary material for: Low-threshold interlayer exciton multiplication in twisted transition metal dichalcogenides heterobilayers
Source: Light Sci Appl. 2026 Feb 10;15:113. doi: 10.1038/s41377-026-02193-w (PMC12886789; doi:10.1038/s41377-026-02193-w)
Supplement: Supplementary file 1 — Supplementary Information [file 41377_2026_2193_MOESM1_ESM.pdf]

Supplementary Information for

Low-threshold interlayer exciton multiplication in twisted transition metal dichalcogenides heterobilayers

Pengzhi Wang[1#], Gan Wang[2#], Chenhao Wang[1#], Qi Wei[1], Yuxuan Chen[2], Qi Liu[1], Luwei Zhou[1], Hui Ren[1], Xiang Zhang[1], Jiangbo Peng[1], Leyi Zhao[1], Tao-Yuan Du[2], Mingjie Li[1,3,4]**

1. Department of Applied Physics, The Hong Kong Polytechnic University, Hung Hom, Kowloon, Hong Kong, China
2. School of Mathematics and Physics, China University of Geosciences, Wuhan 430074, China
3. Shenzhen Research Institute, The Hong Kong Polytechnic University, Shenzhen, Guangdong, 518057, China
4. Photonics Research Institute, The Hong Kong Polytechnic University, Hung Hom, Kowloon, Hong Kong, China

# These authors contributed equally to this work

*Email: ming-jie.li@polyu.edu.hk; duty@cug.edu.cn

Catalogue

Supplementary Note 1 | Quantification of interlayer multiple generation efficiency
Supplementary Note 2 | Raman and steady-state absorption spectra
Supplementary Note 3 | Interlayer charge transfer, IX formation and recombination dynamics
Supplementary Note 4 | Maximum PCE of a single junction photovoltaic device with IXM
Supplementary Note 5 | Mechanism of photon-energy dependence of interlayer exciton multiplication efficiency
Supplementary Note 6 | Multiple interlayer excitons dynamics below threshold under high-fluence conditions
Supplementary Fig. 1 | Optical images and SHG measurements
Supplementary Fig. 2 | Raman and PL spectra
Supplementary Fig. 3 | Calculated electron and hole partial charge density distributions
Supplementary Fig. 4 | Absorption spectra
Supplementary Fig. 5 | Microscopic broadband ultrafast transient absorption spectroscopy setup
Supplementary Fig. 6 | TA spectra of the other twisted heterobilayers
Supplementary Fig. 7 | TA spectra and photocarriers dynamics
Supplementary Fig. 8 | Decay dynamics
Supplementary Fig. 9 | PCEs and IXM efficiency
Supplementary Fig. 10−17 | Absorbed fluence dependence of electron/hole dynamics in IXs of the MoS_2/WSe_2 heterobilayer
Supplementary Fig. 18 | IXM efficiency fitting
Supplementary Fig. 19 | Rise dynamics
Supplementary Fig. 20 | Fitted rise time
Supplementary Fig. 21 | Optical characterization of the $MoTe_2/MoS_2$ heterobilayer
Supplementary Fig. 22 | Interlayer exciton multiplication in the $MoTe_2/MoS_2$ heterobilayer
Supplementary Fig. 23 | Absorbed fluence dependence of electron/hole dynamics in IXs of the $MoTe_2/MoS_2$ heterobilayer
Supplementary Fig. 24 | Density of trion states
Supplementary Fig. 25 | TA spectroscopy of WSe_2 monolayer under sub-threshold energy excitation conditions with high absorbed fluences
Supplementary Fig. 26 | TA spectroscopy of MoS_2/WSe_2 heterobilayer under sub-threshold energy excitation conditions with high absorbed fluences
Supplementary Fig. 27 | Redshift of TA peaks of twisted MoS_2/WSe_2 heterobilayer
Supplementary Fig. 28 | Peak energy versus quantum yield
Supplementary Fig. 29−30 | I−V characteristics
Supplementary Fig. 31 | IQE, EQE and responsivity
Supplementary Table 1 | DFT calculated fractions of the carrier distribution
Supplementary Tables 2–5| Decay lifetime and amplitudes of heterobilayers.
Supplementary Table 6 | Fitted parameters by the Lennard-Jones potential

Supplementary Note 1 | Quantification of interlayer exciton multiplication efficiency

The ideal IXM quantum yield is: $\mathrm{QY_{ideal}} = m = \lfloor h\nu/E_g \rfloor$, where m represents the number of excitons generated by an absorbed photon $h\nu$, with the $\lfloor\ \rfloor$ operator signifying rounding down to the nearest integer. When considering the interplay between hot carrier cooling and IXM processes, the QY model can be depicted as:[1]

$$\mathrm{QY_{IXM}} = \sum_{j=1}^{m} j \frac{k_{\mathrm{cool}} \prod_{i=1}^{j} \kappa_{\mathrm{IXM}}^{(i-1)}}{\prod_{i=1}^{j}(\kappa_{\mathrm{cool}} + \kappa_{\mathrm{IXM}}^{(i)})} \quad (1)$$

where κ_{cool} is the cooling rate, κ_{IXM} is the multiple exciton generation rate, and $\kappa_{\mathrm{IXM}}^{1}=0$ when $h\nu < 2E_{\mathrm{g}}$. The competition between κ_{IXM} and κ_{cool} is formulated as:

$$\kappa_{\mathrm{IXM}}^{(m)} = \kappa_{\mathrm{cool}} P\left(\frac{h\nu - h\nu_{\mathrm{th}}^{(m)}}{h\nu_{\mathrm{th}}}\right)^{s} \theta(h\nu - h\nu_{\mathrm{th}}^{(m)}) \quad (2)$$

Here, P represents the competition factor between κ_{IXM} and κ_{cool}, θ denotes the Heaviside step function, and in the original Keldysh treatment for an ideal semiconductor, s is assigned a value of 2, correlating with the alterations in how κ_{IXM} fluctuates with escalating excess energy. Finally, IXM efficiency (η_{IXM}) can be defined as:

$$\eta_{\mathrm{IXM}} = \frac{P}{P+1} \quad (3)$$

Referring to the model outlined above, for simplification, $\mathrm{QY_{IXM}} \approx 1 + \frac{1}{\tau_{\mathrm{IXM}}/\tau_{\mathrm{cool}}+1}$, when $\tau_{\mathrm{IXM}} \ll \tau_{\mathrm{cool}}$, the QY approaches approximately 200% within the range of $2E_{\mathrm{g}} < h\nu < 3E_{\mathrm{g}}$. Conversely, when $\tau_{\mathrm{IXM}} \approx \tau_{\mathrm{cool}}$, the QY tends to reach around 150%. If $\tau_{\mathrm{IXM}} > \tau_{\mathrm{cool}}$, the QY falls below 150%.

Supplementary Note 2 | Raman and steady-state absorption spectra

Raman spectroscopy confirms the structural integrity and interlayer coupling of the heterobilayers. In monolayer WSe_2 (Supplementary Fig. 2a), the peaks near 250 cm^{-1} correspond to the in-plane (E^1_{2g}) and out-of-plane (A_1g) phonon modes, consistent with previous reports[2]. For monolayer MoS_2, the characteristic peaks at 385.5 cm^{-1} (E^1_{2g}) and 403.9 cm^{-1} (A_1g), separated by 18.4 cm^{-1}, confirm its monolayer nature[3]. The heterobilayer spectrum reveals two new out-of-plane modes: an E'' mode at 284 cm^{-1} (MoS_2-derived) and an A^1_{2g} mode at 309 cm^{-1} (WSe_2-derived), as shown in the inset of Supplementary Fig. 2a. The emergence of these modes is a distinctive signature of strong interlayer coupling, which is present across all twist angles studied[4-6].

To quantify the absorbed photon density, we measured the steady-state micro-area absorption spectra of the individual monolayers and the heterobilayers (Supplementary Fig. 4). Monolayer MoS_2 exhibits three distinct excitonic peaks (A, B, C), while monolayer WSe_2 shows four (A, B, C, D). The A and B excitons in both materials originate from transitions between the spin-orbit split valence bands and the conduction band at the K points of the Brillouin zone[7-10]. The higher-energy C and D excitons, which exhibit strong absorption due to a nesting effect[11, 12], arise from transitions between the Γ and K points[13, 14]. In the heterobilayers, the absorption spectrum is a superposition of the monolayer features, though the peaks are broadened due to charge transfer effects[15].

Supplementary Note 3 | Interlayer charge transfer, IX formation and recombination dynamics

The carrier dynamics in the monolayer regions of the heterobilayer (Supplementary Fig. 7a-c; panel c mirrors Fig. 2c on a shorter timescale) show a rapid signal rise, well-modeled by a Gaussian function with a 0.2 ps full width at half maximum. This rise time is consistent with previous pump-probe studies of monolayer TMDs[16, 17] and indicates that the initial carrier population is established faster than our instrumental resolution. In the heterobilayer region, the signals probed at the MoS_2 (M probe) and WSe_2 (W probe) band edges exhibit slower rise times of 0.3 ps and 0.4 ps, respectively. The additional ~0.1-0.2 ps delay compared to the monolayers is attributed to interlayer charge transfer and the formation of interlayer excitons (IXs).

The decay dynamics of the monolayer MoS_2 and WSe_2 signals are fitted with a biexponential function, $\Delta T/T_0(t) = A_1 * \exp(-t/\tau_1) + A_2 * \exp(-t/\tau_2)$. The fast decay component ($\tau_1 \approx 1.30 \pm 0.10$ ps for MoS_2; 0.54 ± 0.05 ps for WSe_2) corresponds to intralayer exciton formation mediated by strong Coulomb attraction[18-20]. The subsequent slow decay ($\tau_2 \approx 40.30 \pm 1.70$ ps for MoS_2; 24.28 ± 1.20 ps for WSe_2) arises from non-radiative exciton recombination[16, 21].

In stark contrast, the probe signals from the heterobilayer exhibit a long-lived decay extending into the nanosecond regime. This pronounced difference confirms efficient charge separation and the formation of stable interlayer excitons, consistent with the type-II band alignment.

We further analyzed the electron and hole decay dynamics in the twisted heterobilayers using a biexponential model (Supplementary Fig. 8 and Tables 2-5). The fast decay component (< 5 ps) observed for IX electrons (probed at the MoS_2 band edge) is attributed to Coulomb scattering with hybrid holes at the Γ point. The long-lived electron decay originates from the recombination of both K–K and K–Γ IXs. Conversely, the hole dynamics (probed at the WSe_2 band edge) are governed by two longer time constants associated solely with the recombination of K–K IXs, the lifetimes of which were determined using a weighted average.

Supplementary Note 4 | Maximum PCE of a single junction photovoltaic device with IXM

The power conversion efficiency (PCE) limit of a single-junction IXM photovoltaic device is determined using the detailed balance model proposed by Shockley and Quisser as follows[22, 23]:

$$\mathrm{PCE} = V \times \frac{j_{\mathrm{ext}}(E_{\mathrm{g}},V)}{P_{\mathrm{s}}} \tag{4}$$

Here, P_{s} represents the incident power from AM 1.5G solar irradiation. The external current, j_{ext}, is derived as the disparity between the absorbed spectral photon flux, j_{abs}, of the AM 1.5G solar irradiance and the emitted photon flux, j_{em}, at an external voltage *V*:

$$j_{\mathrm{ext}}(E_{\mathrm{g}},V) = \int_{E_{\mathrm{g}}}^{\infty} dE[j_{\mathrm{abs}}(E_{\mathrm{g}}) - j_{\mathrm{em}}(E_{\mathrm{g}},V)] \tag{5}$$

The absorbed photon flux is ascertained by:

$$j_{\mathrm{abs}}(E) = \frac{q\lambda \mathrm{QY}_{\mathrm{IXM}}}{hc} \mathrm{AM1.5G} \tag{6}$$

where *q* represents the charge unit, λ the light wavelength, *h* the Plank constant, *c* the speed of light, β the multiple exciton generation yield, and E_{th} the IXM threshold. In the ideal IXM process, the quantum yield of IXM ($\mathrm{QY}_{\mathrm{IXM}}$) adheres to a step function. This means that when the photon energy attains N^*E_{g}, the maximum IXM QY is N. The emitted photon flux, j_{em}, is computed utilizing the generalized Planck's law:

$$j_{\mathrm{em}}(E,V) = \frac{q2\pi E^2}{h^3c^2} \frac{1}{[\exp\left(\frac{\mathrm{E-qV}}{\kappa_{\mathrm{B}} T}\right) - 1]} \tag{7}$$

where κ_{B} is the Boltzmann constant.

Supplementary Note 5 | Mechanism of photon-energy dependence of interlayer exciton multiplication efficiency

As shown in Supplementary Fig 18a and b, when comparing the measured quantum yield (QY) and IX multiplication efficiency (η_{IXM}) curves, η_{IXM} for IX for both IX electrons and holes decreases with increasing photon energy. IX multiplication (IXM) depends on the ratio of carrier scattering efficiency (W_X) to cooling efficiency (γ_{cool}), a ratio that varies with excitation photon energy. As illustrated in Fig. 4a, this relationship is governed by the energy-dependent scattering pathways of carriers in the conduction band (for electrons) and valence band (for holes), respectively. Consequently, differences in the involved energy bands, dispersion relations, density of states, and momentum-matching mechanisms for these scattering pathways result in distinct dependencies of the QYs of interlayer exciton electron multiplication (IEM) and interlayer exciton hole multiplication (IHM) on excitation photon energy.

To gain numerical insight, we note from Eq. (1) in the main text that the multiplication processes of different carriers are coupled, making it non-trivial to directly resolve how carrier scattering efficiency and cooling efficiency vary with photon energy. We therefore introduce an approximation: although electrons and holes each account for only a fraction of the light absorption channels, we assume the total absorption spectrum $D(h\nu)$ can be approximated as:

$$D(h\nu) = \frac{1}{N_k^2}\sum_X D_X(h\nu) \tag{8}$$

This approximation captures the material's absorption spectrum, as shown in Supplementary Fig. 4. Under this assumption, we can define an effective ratio of the carrier-carrier scattering rate to the intralayer cooling rate, given by:

$$\langle\frac{W_X}{\gamma_{\mathrm{cool}}}\rangle(h\nu) = \left[\left(\frac{\mathrm{QY}(h\nu)}{D(h\nu)} - 1\right)^{-1} - 1\right]^{-1} \tag{9}$$

By combining above equation with QY vs $h\nu$ data in the main text, we can extract the dependence of $\langle\frac{W_X}{\gamma_{\mathrm{cool}}}\rangle(h\nu)$ (Supplementary Fig. 18, c and d). The results show that the hot-carrier scattering strength decreases relative to hot-carrier cooling at higher photon energies.

First, from the twist-angle dependence of η_{IXM}, and considering the weak twist-angle dependence of intralayer hot-carrier cooling (as reflected by the similar rise times below the IXM threshold; Supplementary Fig. 20e, f), we can deduce that the reduction in $\langle\frac{W_X}{\gamma_{\mathrm{cool}}}\rangle$ at larger twist angles is more likely due to diminished interlayer carrier-carrier scattering. Therefore, the decrease in η_{IXM} under very high photon energy excitation could be associated with a reduced density of states at higher energy levels involved, which weakens interlayer carrier-carrier scattering.

Supplementary Note 6 | Multiple interlayer excitons dynamics below the $2E_{\mathrm{g(type-II)}}$ threshold under high-fluence conditions

A pump pulse with energy above the MoS_2 bandgap (1.85 eV) can generate carriers in both monolayers. Due to the type-II band alignment, electrons from WSe_2 transfer to the MoS_2 conduction band, while holes from MoS_2 transfer to the WSe_2 valence band (forming K–K IXs) or the hybridized Γ-point (forming K–Γ IXs). These populations are monitored by probe pulses at the A-exciton resonances: probing MoS_2 detects electrons from both K–K and K–Γ IXs, while probing WSe_2 selectively monitors holes from K–K IXs.

To investigate high-fluence dynamics below the IXM threshold, we used a 1.90 eV pump and varied the fluence. A redshift of the transient absorption peaks for both MoS_2 and WSe_2 emerges only at high fluence ($>20 \times 10^{12}$ cm^{-2}, Supplementary Fig. 27a). This is consistent with the attractive interactions observed between multiple IXs generated *above* the threshold at low fluence (3.0×10^{12} cm^{-2}) via quantum-exchange correlations (Fig. 5).

The carrier dynamics at various fluences are shown in Supplementary Figs. 27b (electrons) and 27c (holes). The peak signal exhibits a sublinear, saturable dependence on fluence (Supplementary Fig. 27d), a common effect in 2D semiconductors at high carrier densities.

Fitting the dynamics with bi-exponential functions (solid lines in Supplementary Figs. 27b, c) yields the recombination lifetimes for K–K/K–Γ electrons and K–K holes. These lifetimes are compiled in Supplementary Fig. 27e and remain constant across fluence, despite carrier densities an order of magnitude higher than in the main-text experiments. This fluence independence confirms that multiple interlayer excitons are inherently long-lived, even under high-density excitation below the multiplication threshold.

For comparison, high-fluence measurements on a WSe_2 monolayer with the same 1.90 eV pump show no peak redshift (Supplementary Fig. 28a), confirming that the redshift in the heterobilayer is a unique property of the spatially separated IXs. The monolayer dynamics are also biexponential (Supplementary Fig. 28b), with the longer component representing exciton recombination. In contrast to the stable IXs, the intralayer excitons in WSe_2 exhibit pronounced fluence-dependent decay (Supplementary Fig. 28d), a signature of exciton-exciton annihilation due to strong many-body interactions in the monolayer.

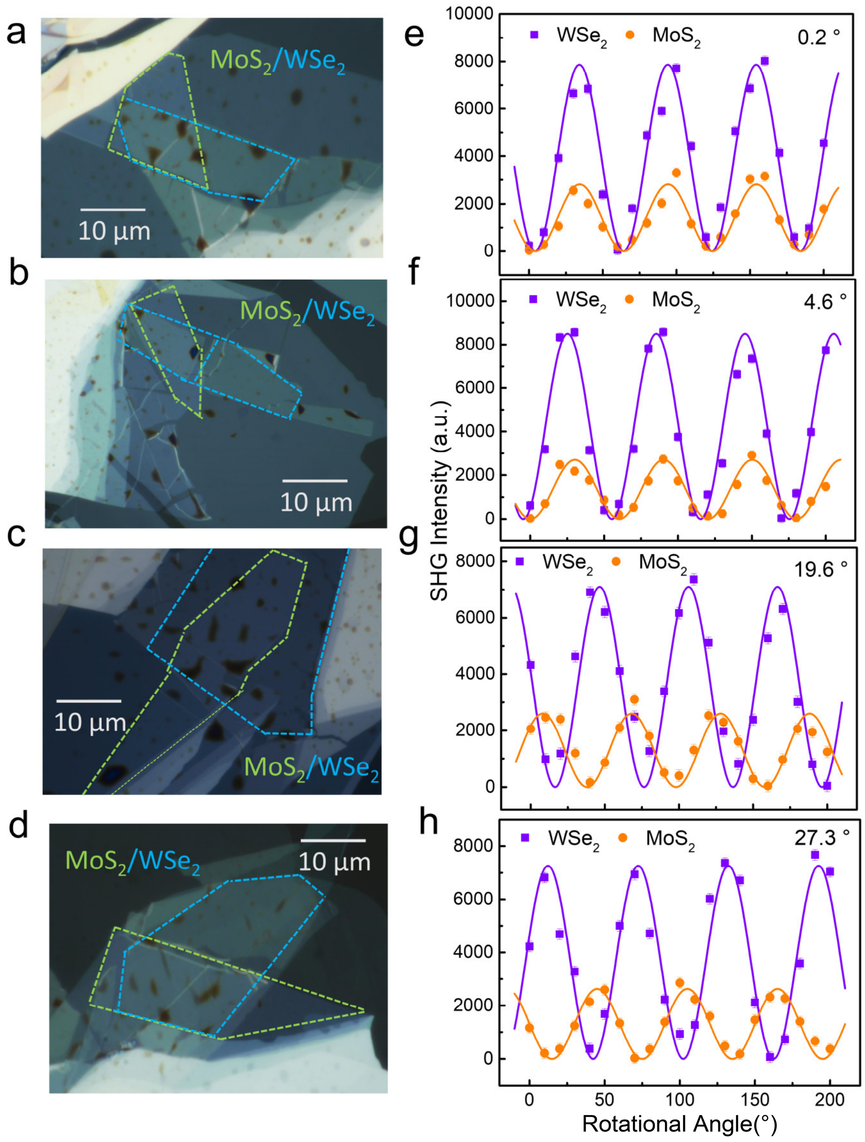

Supplementary Fig. 1 | Optical images and SHG measurements. a–d, Optical microscope images of h-BN capped twisted MoS_2/WSe_2 heterobilayers on quartz substrates. **e–h,** Second harmonic generation (SHG) intensity from the monolayer regions of WSe_2 and MoS_2 as a function of the crystal's rotational angle after stacking. The solid lines are the corresponding fits, which confirm the twist angles of 0.2 ± 0.5°, 4.6 ± 0.5°, 19.6 ± 0.5° and 27.3 ± 0.5°, between MoS_2 and WSe_2 layers.

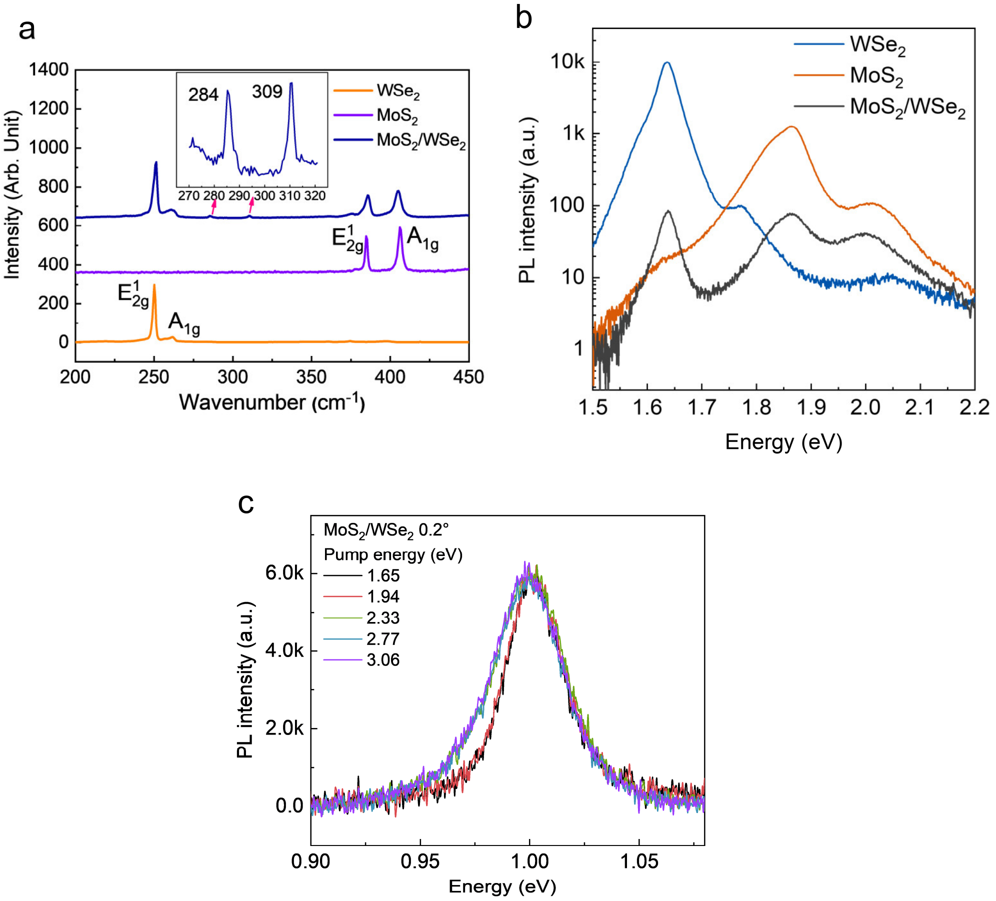

Supplementary Fig. 2 | Raman and PL spectra. a, Raman spectra of the MoS_2 ML, WSe_2 ML and MoS_2/WSe_2 heterobilayer with twist angle of 0.2° under 2.33 eV excitation. Inset provides a closer view around 300 cm^{-1}. **b,** Room-temperature PL spectra of the ML regions at MoS_2 and WSe_2 and MoS_2/WSe_2 heterobilayer region. **c**, Normalized interlayer exciton emission with various excitation energies at room temperature. As observed, a weak emission on the lower-energy side emerges for excitations above the $2E_{\mathrm{g(type-II)}}$ threshold. This can be attributed to trion formation via the binding of a charge carrier to an IX, a process with relatively low probability. Since only a fraction of multiple IXs converts to trions, the measured IX efficiency of ~ 90% from PL (consistent with Fig. 1e) is thus reasonable. This behavior is consistent with our main text claim that multiple IXs are generated when the incident pump photon energy exceeds the $2E_{\mathrm{g(type-II)}}$ threshold.

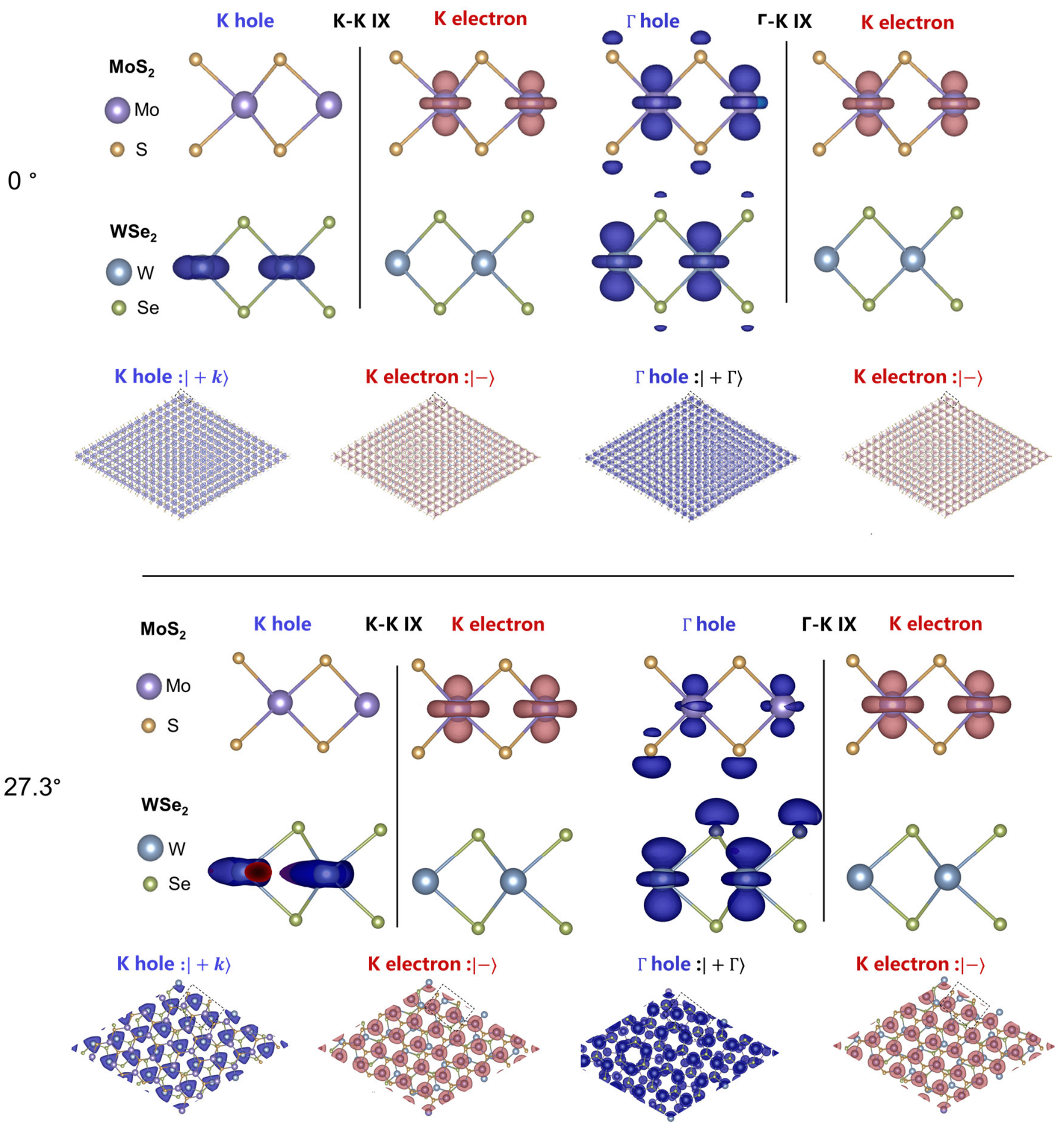

Supplementary Fig. 3 | Calculated electron and hole partial charge density distributions in 0° (top) and 27.3° (bottom) stacked MoS_2/WSe_2 heterobilayers with cross-sectional and top-down views. The fractions of carrier distribution are listed in Table S1.

Supplementary Table 1. DFT calculated fractions of the carrier distribution in each layer of MoS_2/WSe_2 heterobilayers.

**0°**	**Γ hole**	**K hole**	**K electron**
**WSe_2**	76%	100%	0%
**MoS_2**	24%	0%	100%
**27.3°**	**Γ hole**	**K hole**	**K electron**
**WSe_2**	69%	98%	1%
**MoS_2**	31%	2%	99%

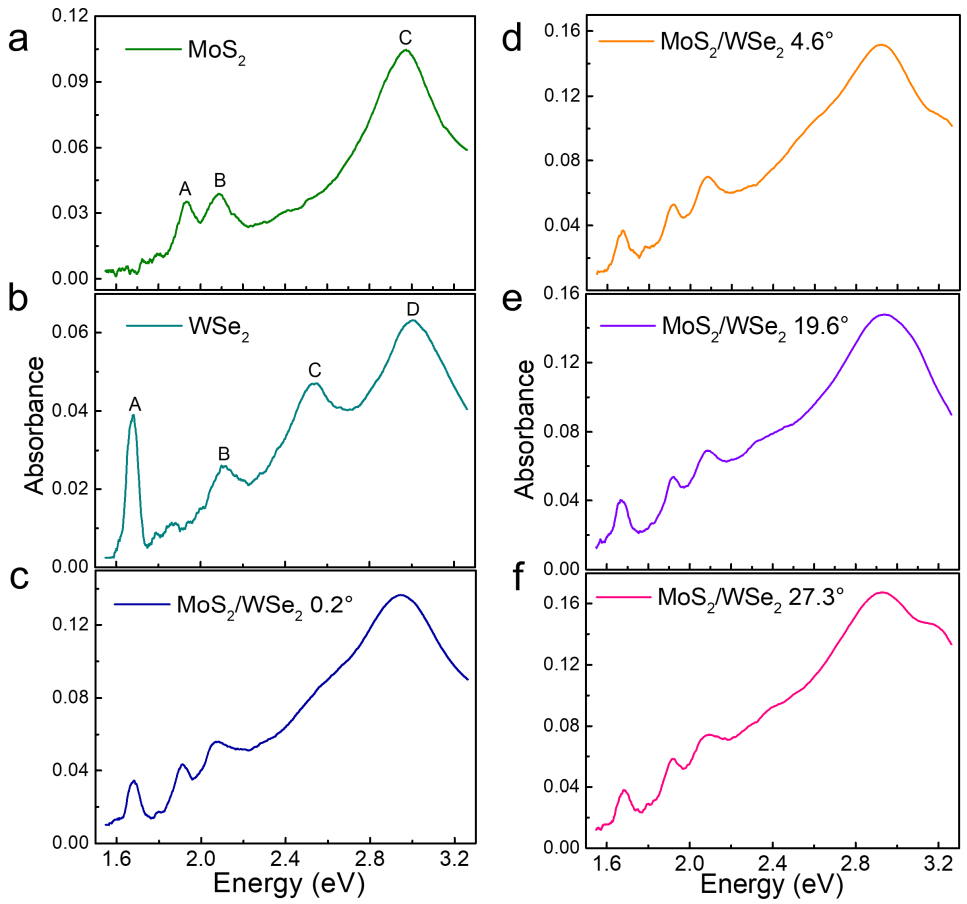

Supplementary Fig. 4 | Absorption spectra. a–f, Steady-state absorption spectra of ML MoS_2, ML WSe_2 and the stacked MoS_2/WSe_2 heterobilayers, with twist angles varying between 0° and 30° as labeled.

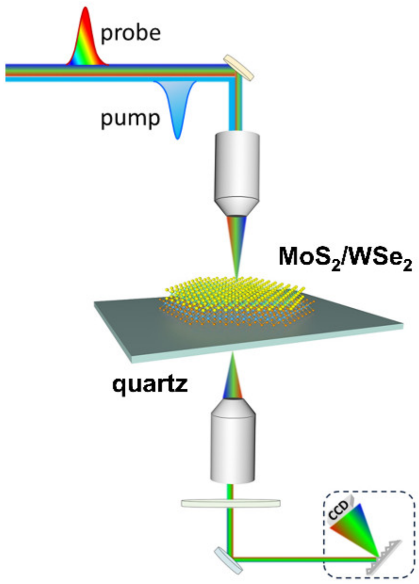

Supplementary Fig. 5 | Microscopic broadband ultrafast transient absorption spectroscopy setup. We excited the samples with an ultrafast laser pulse at a specific photon energy ($h\nu_{ex}$). Following a set delay time, we assessed the relative change ($\Delta T/T$) of a transmitted white light continuum probe that encompasses the visible and near-IR spectrum.

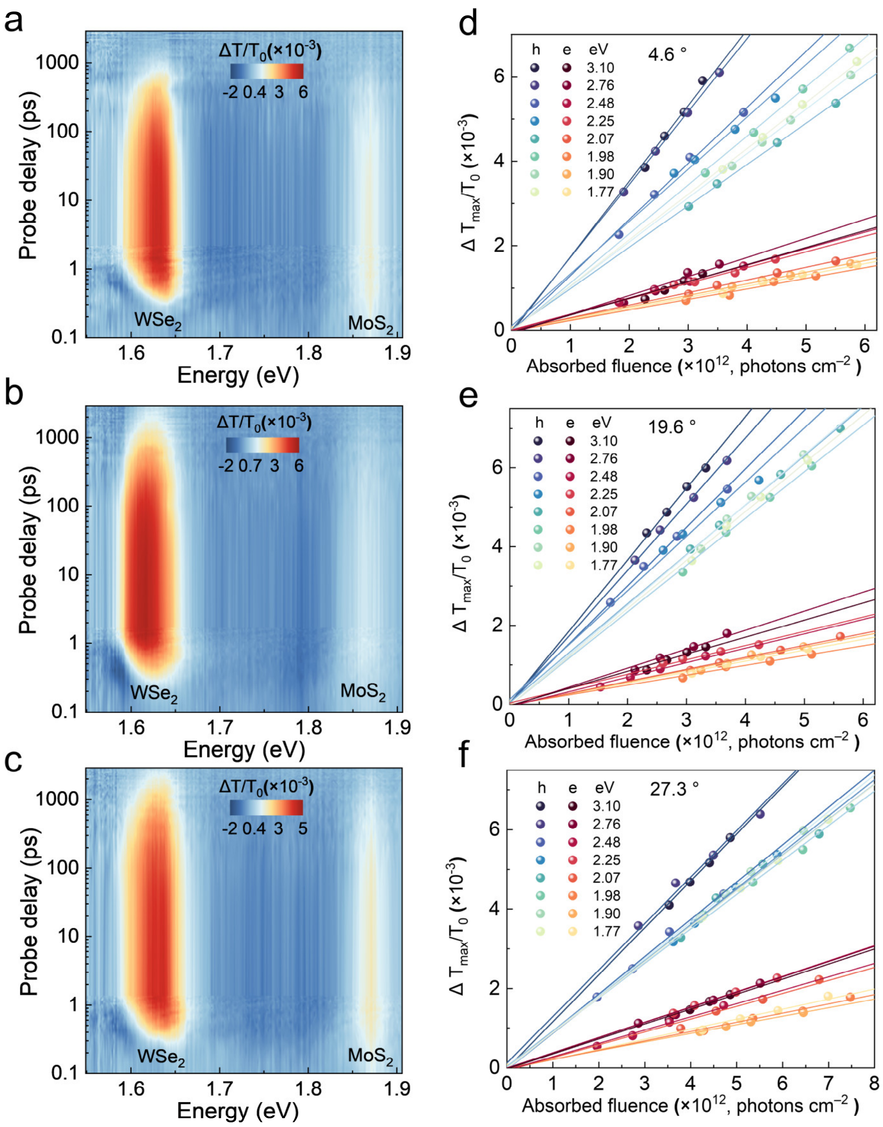

Supplementary Fig. 6 | TA spectra of the other twisted heterobilayers. a−c, 2D color plot of the transient absorption (TA) spectra of the MoS_2/WSe_2 heterobilayer, with 4.6°, 19.6°and 27.3° twist angles, respectively, under the excitation provided by a 2.48 eV pump pulse. **d−f,** The maximum $\Delta T_{\max}/T_0$ intensity as a function of the absorbed fluence at various pump photon energies for the corresponding heterobilayer with respective twist angle as labeled. The solid lines in the plot correspond to linear fits, and the slope of these lines indicates the quantum yield (QY).

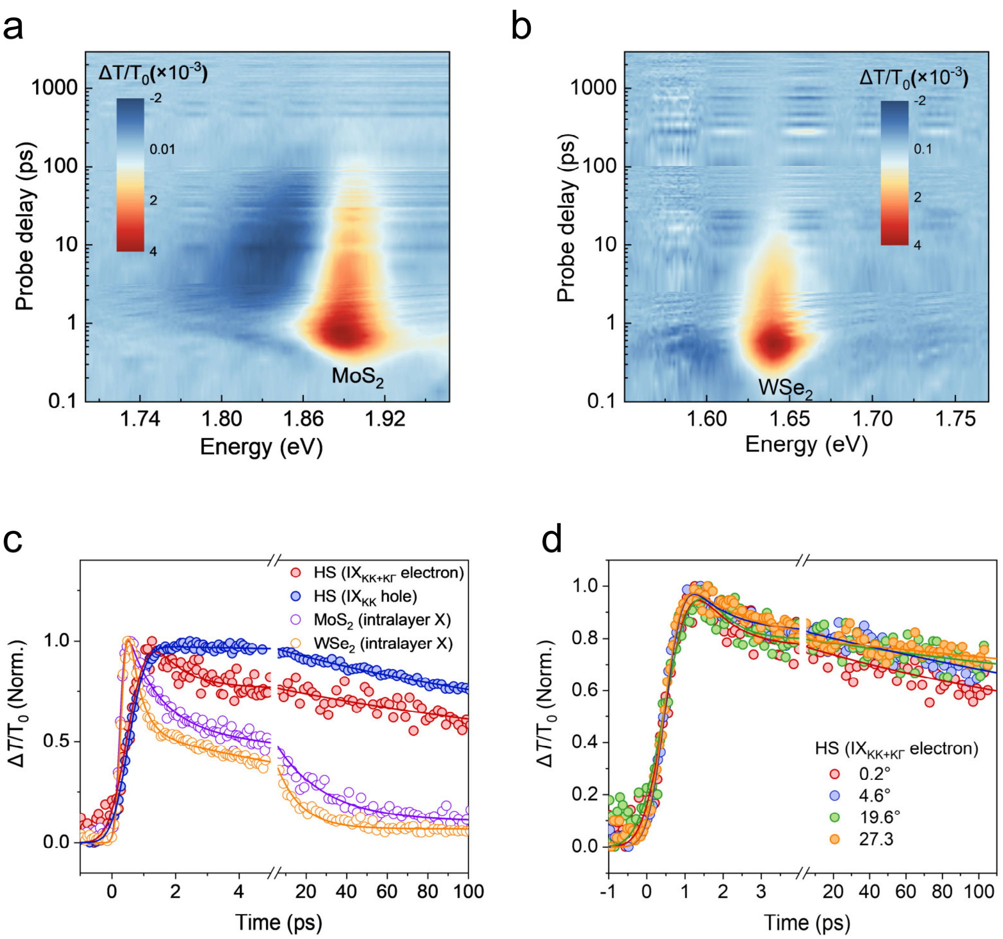

Supplementary Fig. 7 | TA spectra and photocarriers dynamics. a-b, 2D color plots of the transient absorption (TA) spectra of monolayer MoS_2 and monolayer WSe_2, respectively, under 2.48 eV photon excitation. **c,** Normalized TA dynamics on a timescale below 100 ps for MoS_2 and WSe_2 MLs, and their heterobilayer with twist angle of 0.2° under 2.48 eV photon excitation. **d,** Normalized TA dynamics of electrons in IX on a timescale below 100 ps of twisted heterobilayers under 2.48 eV photon excitation.

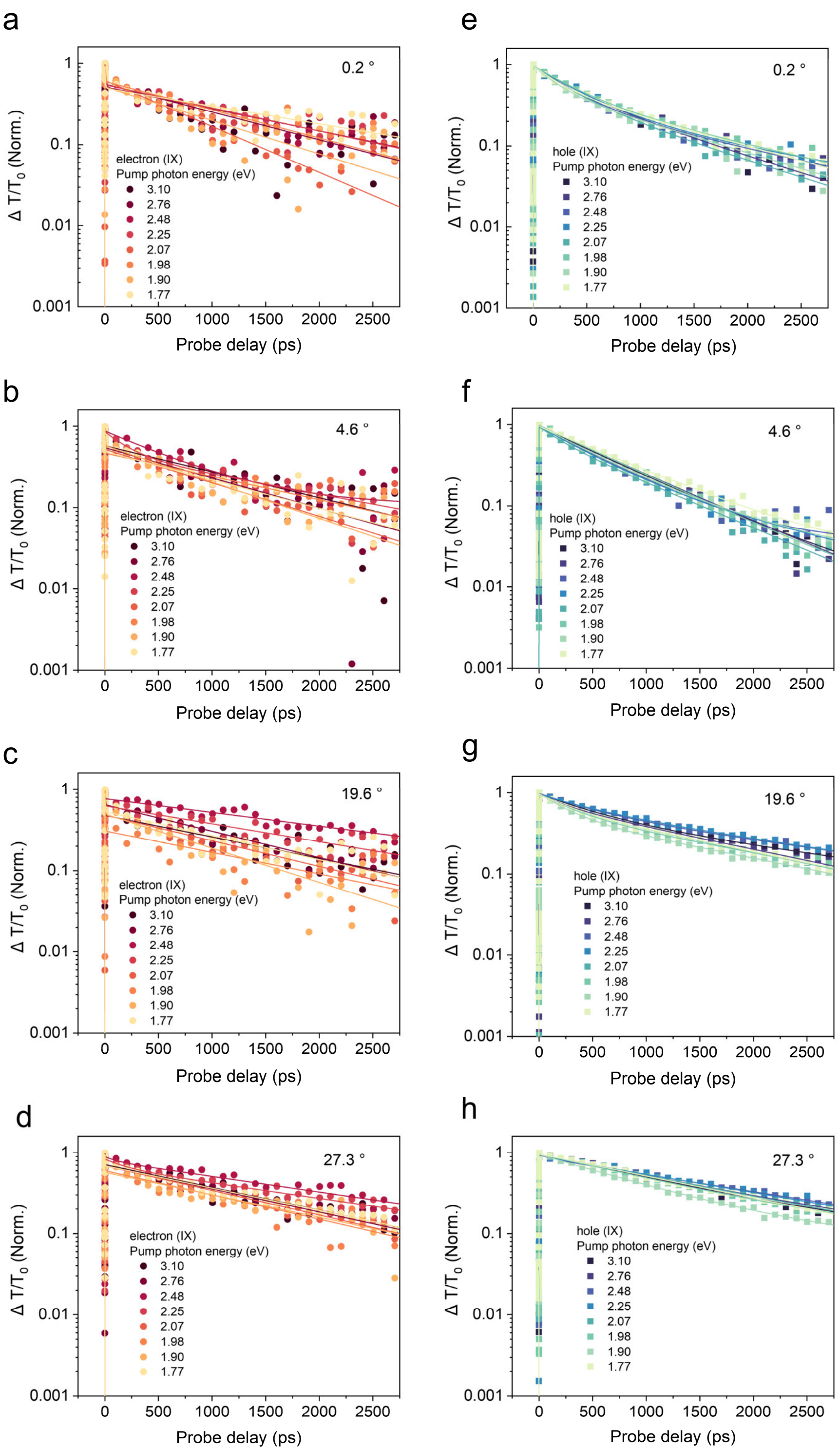

Supplementary Fig. 8 | IX recombination dynamics. a−h, Normalized TA dynamics probed at MoS_2 (**a−d**) and WSe_2 (**e−h**) transitions in the 0.2°, 4.6°, 19.6° and 27.3° MoS_2/WSe_2 heterobilayers, respectively. The fitted lifetime and amplitudes are provided in Table S2-S5.

Supplementary Table 2. Decay lifetime and amplitudes of 0.2° heterobilayers

0.2°	electron (IX)		hole (IX)		
Pump energy (eV)	τ_1 (A_1) [ps]	τ_2 (A_2) [ps]	τ_1 (A_1) [ps]	τ_2 (A_2) [ps]	$A_1\tau_1 + A_2\tau_2$ [ps]
3.10	4.2 ± 1.0 (57%)	1353 ± 120 (43%)	666 ± 105 (93%)	1701 ± 698 (7%)	738 ± 147
2.76	3.3 ± 0.7 (47%)	1266 ± 134 (53%)	529 ± 121 (31%)	820 ± 133 (69%)	730 ± 129
2.48	4.2 ± 1.0 (47%)	1538 ± 157 (53%)		618 ± 31 (100%)	618 ± 31
2.25	4.6 ± 0.8 (45%)	1528 ± 146 (55%)	506 ± 80 (78%)	1541 ± 374 (22%)	734 ± 145
2.07	3.5 ± 0.9 (45%)	1068 ± 70 (55%)	213 ± 70 (22%)	752 ± 111 (78%)	634 ± 102
1.98	3.4 ± 0.7 (41%)	1248 ± 112 (59%)		715 ± 26 (100%)	715 ± 26
1.90	4.9 ± 1.5 (49%)	1195 ± 176 (51%)	584 ± 98 (89%)	2312 ± 501 (11%)	774 ± 142
1.77	4.7 ± 1.0 (48%)	1479 ± 116 (52%)	677 ± 82 (85%)	1289 ± 164 (15%)	768 ± 94
Average value	4.1 ± 1.1 (47%)	1334 ± 129 (53%)			714 ± 102 (100%)

Supplementary Table 3. Decay lifetime and amplitudes of 4.6° heterobilayers

**4.6°**	**electron (IX)**		**hole (IX)**		
Pump energy (eV)	τ_1 (A_1) [ps]	τ_2 (A_2) [ps]	τ_1 (A_1) [ps]	τ_2 (A_2) [ps]	$A_1\tau_1 + A_2\tau_2$ [ps]
3.10	3.6 ± 0.7 (45%)	1359 ± 131 (55%)	365 ± 92 (50%)	1069 ± 154 (50%)	717 ± 123
2.76	2.8 ± 0.5 (50%)	1166 ± 108 (50%)	347 ± 56 (65%)	1500 ± 239 (35%)	751 ± 120
2.48	2.3 ± 0.5 (44%)	1700 ± 156 (56%)	261 ± 51 (55%)	1162 ± 185 (45%)	667 ± 111
2.25	3.6 ± 0.6 (45%)	1359 ± 110 (55%)	437 ± 100 (73%)	1921 ± 207 (27%)	838 ± 129
2.07	3.9 ± 0.8 (48%)	1056 ± 123 (52%)	414 ± 75 (75%)	1609 ± 258 (25%)	713 ± 121
1.98	3.8 ± 0.7 (52%)	1518 ± 190 (48%)	391 ± 76 (60%)	1499 ± 167 (40%)	835 ± 112
1.90	4.4 ± 0.8 (40%)	1172 ± 106 (60%)	219 ± 57 (48%)	1107 ± 165 (52%)	681 ± 113
1.77	3.7 ± 0.6 (49%)	1480 ± 132 (51%)	245 ± 60 (46%)	1204 ± 164 (54%)	763 ± 116
Average value	3.5 ± 0.7 (47%)	1351 ± 132 (53%)			746 ± 118 (100%)

Supplementary Table 4. Decay lifetime and amplitudes of 19.6° heterobilayers

19.6°	electron (IX)		hole (IX)		
Pump energy (eV)	τ_1 (A_1) [ps]	τ_2 (A_2) [ps]	τ_1 (A_1) [ps]	τ_2 (A_2) [ps]	$A_1\tau_1 + A_2\tau_2$ [ps]
3.10	3.3 ± 0.4 (56%)	1638 ± 185 (44%)	540 ± 90 (49%)	2432 ± 286 (51%)	1505 ± 190
2.76	4.0 ± 0.8 (38%)	1334 ± 95 (62%)	275 ± 59 (25%)	1580 ± 133 (75%)	1254 ± 115
2.48	2.5 ± 0.9 (38%)	2579 ± 200 (62%)	496 ± 92 (39%)	2413 ± 291 (61%)	1665 ± 213
2.25	3.8 ± 0.8 (39%)	1973 ± 176 (61%)	505 ± 68 (32%)	2255 ± 260 (68%)	1695 ± 199
2.07	4.7 ± 0.7 (51%)	1373 ± 148 (49%)	310 ± 95 (31%)	1587 ± 170 (69%)	1191 ± 147
1.98	3.3 ± 0.6 (58%)	1638 ± 157 (42%)	334 ± 63 (41%)	1762 ± 191 (59%)	1177 ± 139
1.90	3.6 ± 0.5 (52%)	1833 ± 168 (48%)	388 ± 80 (47%)	1976 ± 241 (53%)	1229 ± 165
1.77	3.3 ± 0.4 (45%)	1756 ± 152 (55%)	383 ± 72 (30%)	1798 ± 201 (70%)	1374 ± 162
Average value	3.6 ± 0.6 (47%)	1766 ± 160 (53%)			1386 ± 166 (100%)

Supplementary Table 5. Decay lifetime and amplitudes of 27.3° heterobilayers

27.3°	electron (IX)		hole (IX)		
Pump energy (eV)	τ_1 (A_1) [ps]	τ_2 (A_2) [ps]	τ_1 (A_1) [ps]	τ_2 (A_2) [ps]	$A_1\tau_1 + A_2\tau_2$ [ps]
3.10	1.8 ± 0.4 (38%)	1537 ± 88 (62%)		1405 ± 94 (100%)	1405 ± 94
2.76	2.0 ± 0.4 (40%)	1515 ± 83 (60%)		1524 ± 97 (100%)	1524 ± 97
2.48	4.1 ± 0.5 (42%)	2237 ± 181 (58%)	150 ± 61 (6%)	2060 ± 114 (94%)	1945 ± 111
2.25	3.2 ± 0.6 (33%)	2338 ± 194 (67%)	83 ± 26 (4%)	1923 ± 89 (96%)	1849 ± 87
2.07	3.2 ± 0.5 (32%)	1425 ± 80 (68%)		1611 ± 132 (100%)	1611 ± 132
1.98	3.1 ± 0.5 (42%)	1444 ± 114 (58%)		1558 ± 139 (100%)	1558 ± 139
1.90	4.4 ± 1.2 (38%)	1564 ± 152 (62%)		1624 ± 243 (100%)	1624 ± 243
1.77	3.0 ± 0.5 (35%)	1854 ± 135 (65%)		1543 ± 147 (100%)	1543 ± 147
Average value	3.1 ± 0.6 (37%)	1739 ± 128 (63%)			1632 ± 131 (100%)

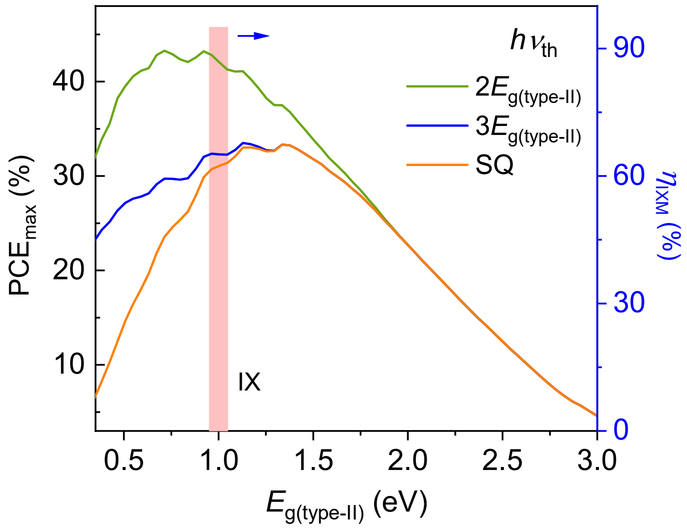

Supplementary Fig. 9 | PCEs and IXM efficiency. Calculated maximum power conversion efficiency (PCEs) (solid lines, see Supplementary Note 4 for details) under AM1.5 solar illumination as a function of $E_{g(type\text{-}II)}$ with different IXM threshold ($h\nu_{th}$) relative to $E_{g(type\text{-}II)}$ and the IXM efficiency (η_{IXM}) of MoS_2/WSe_2 heterobilayer (red column). SQ: Shockley–Queisser limit.

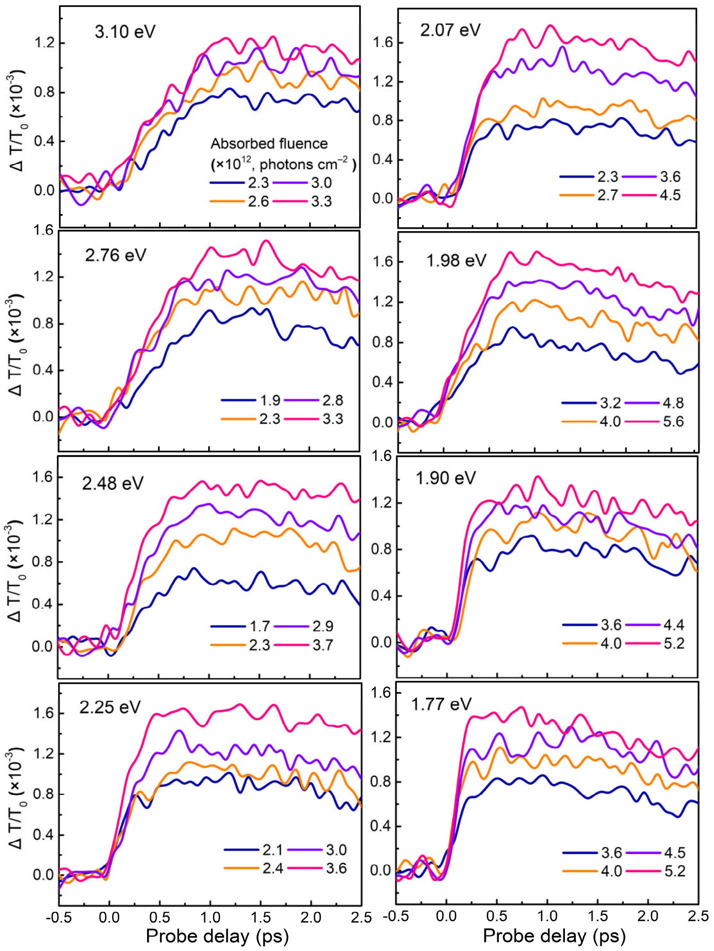

Supplementary Fig. 10 | Absorbed fluence dependence of electron dynamics in IXs of the 0.2° MoS_2/WSe_2 heterobilayers. The probe is tuned to the MoS_2 transition to monitor the electron population of IX.

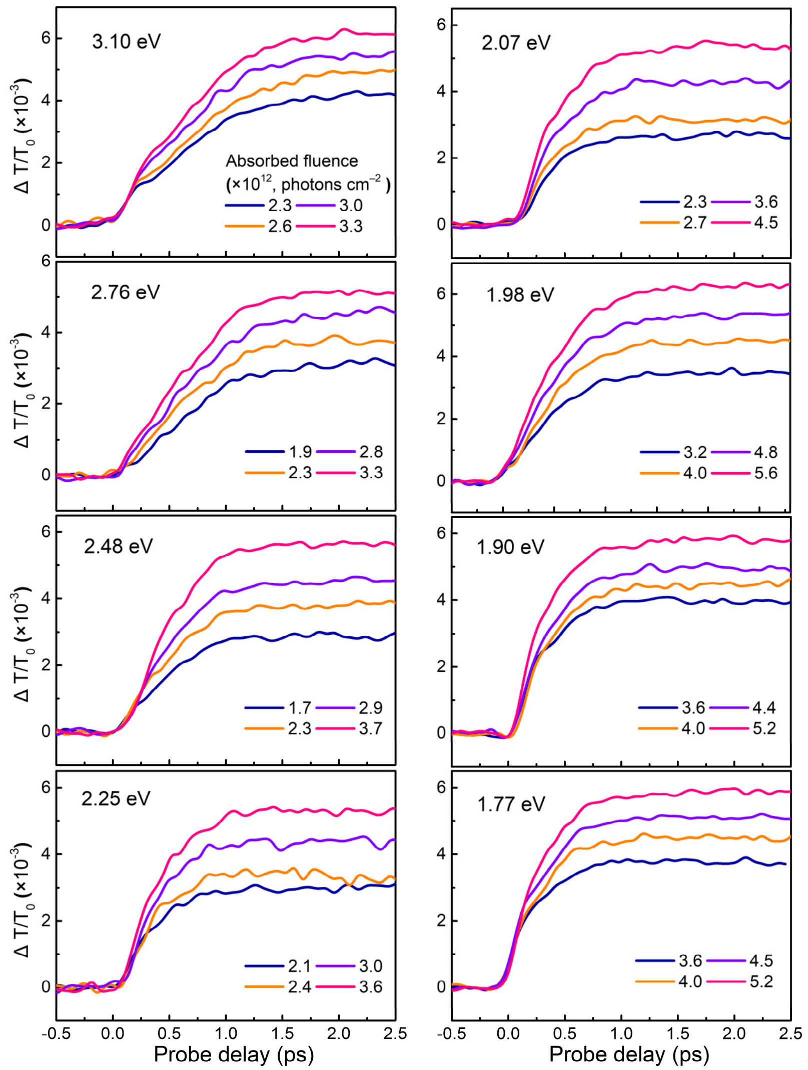

Supplementary Fig. 11 | Absorbed fluence dependence of hole dynamics in IXs of the 0.2° MoS_2/WSe_2 heterobilayers. The probe is tuned to the WSe_2 transition to monitor the hole population of IX.

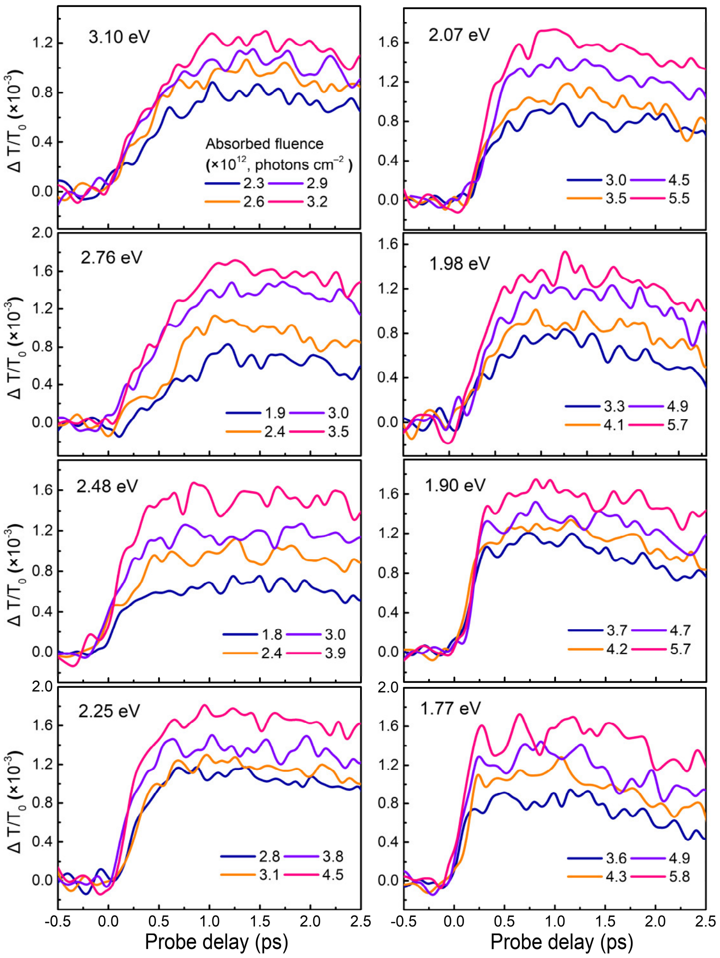

Supplementary Fig. 12 | Absorbed fluence dependence of electron dynamics in IX of the 4.6° MoS_2/WSe_2 heterobilayers. The probe is tuned to the MoS_2 transition to monitor the electron population of IX.

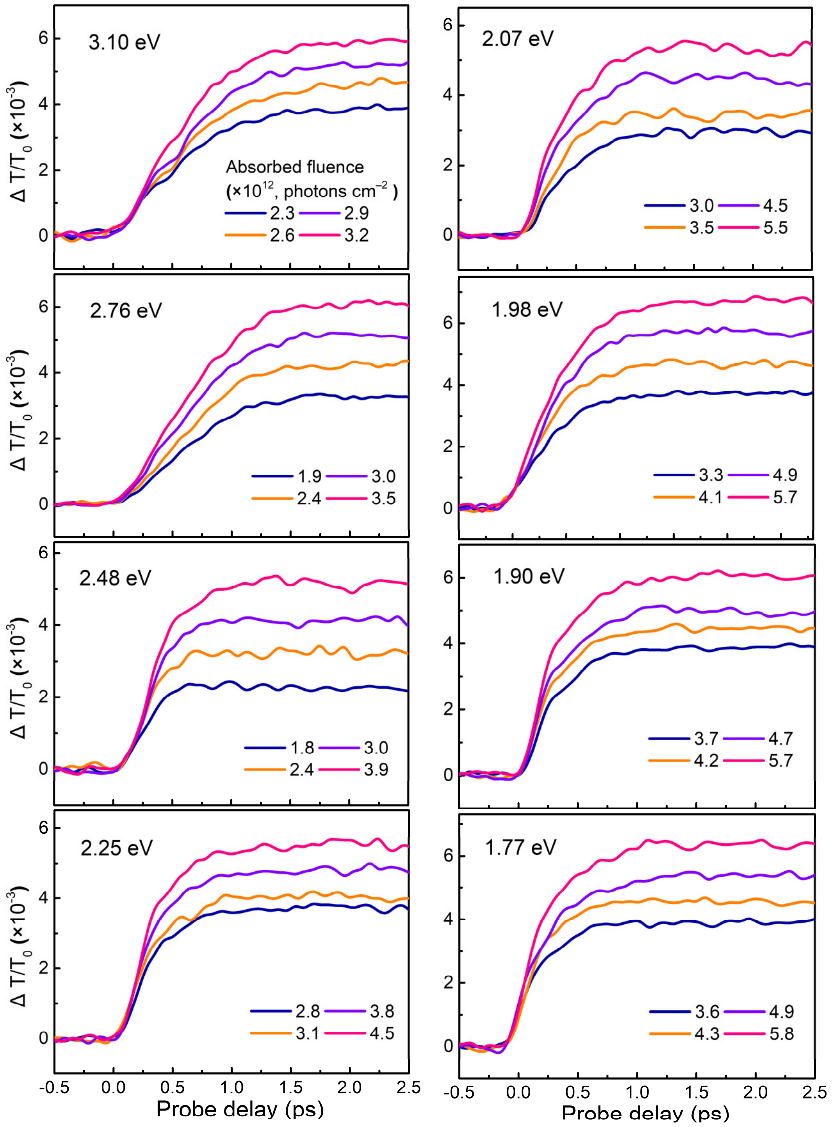

Supplementary Fig. 13 | Absorbed fluence dependence of hole dynamics in IXs of the 4.6° MoS_2/WSe_2 heterobilayers. The probe is tuned to the WSe_2 transition to monitor the hole population of IX.

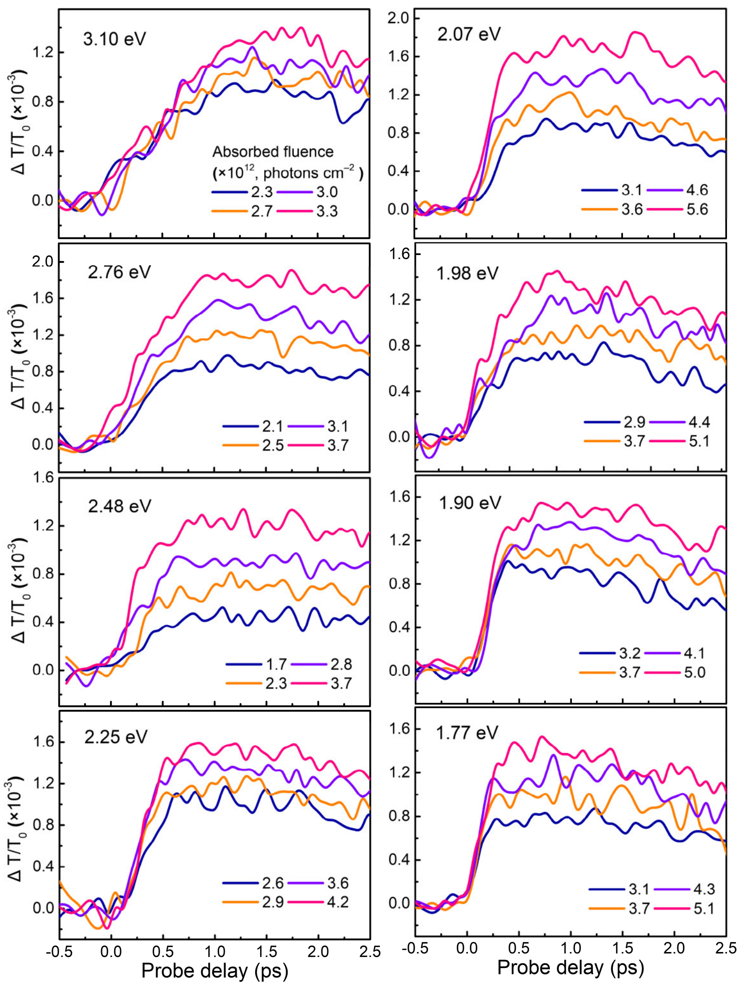

Supplementary Fig. 14 | Absorbed fluence dependence of electron dynamics in IXs of the 19.6° MoS_2/WSe_2 heterobilayers. The probe is tuned to the MoS_2 transition to monitor the electron population of IX.

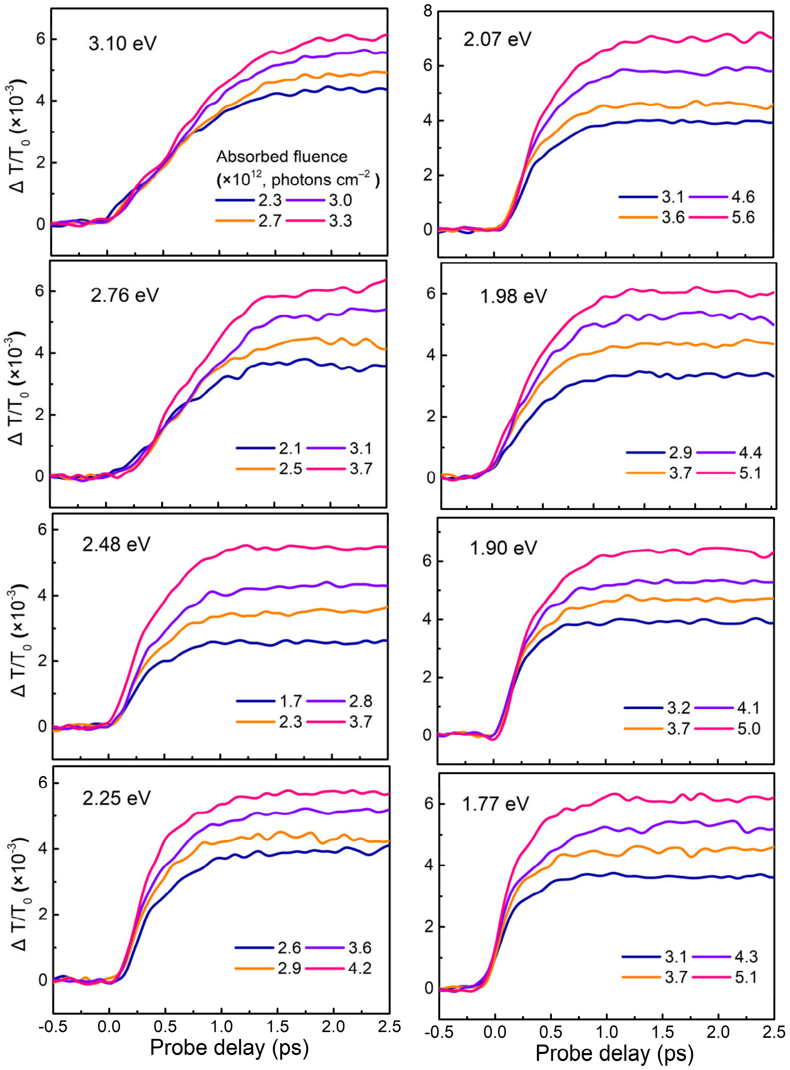

Supplementary Fig. 15 | Absorbed fluence dependence of hole dynamics in IXs of the 19.6° MoS_2/WSe_2 heterobilayers. The probe is tuned to the WSe_2 transition to monitor the hole population of IX.

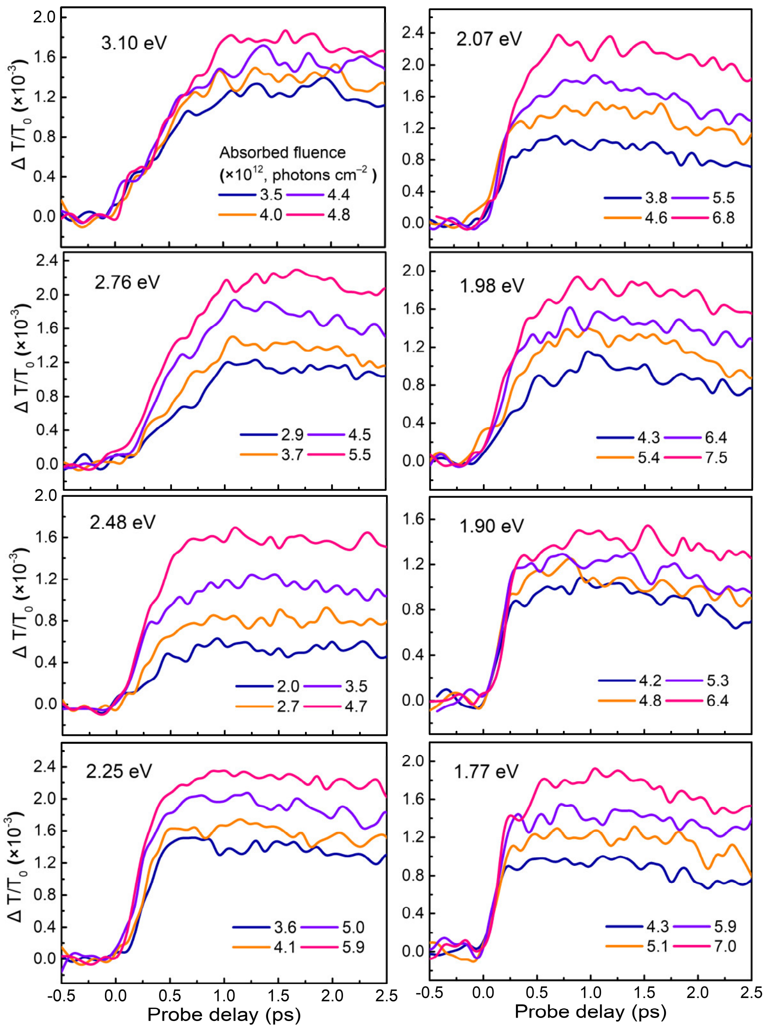

Supplementary Fig. 16 | Absorbed fluence dependence of electron dynamics in IXs of the 27.3° MoS_2/WSe_2 heterobilayers. The probe is tuned to the MoS_2 transition to monitor the electron population of IX.

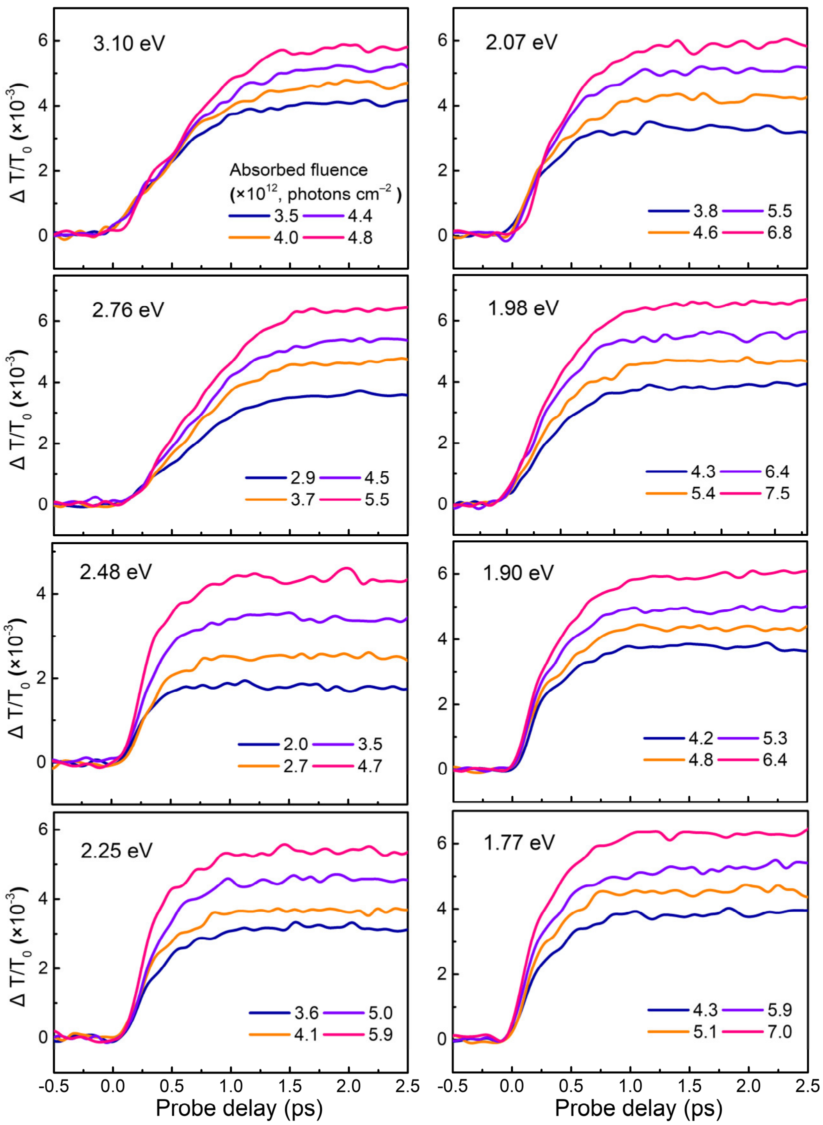

Supplementary Fig. 17 | Absorbed fluence dependence of hole dynamics in IXs of the 27.3° MoS_2/WSe_2 heterobilayers. The probe is tuned to the WSe_2 transition to monitor the hole population of IX.

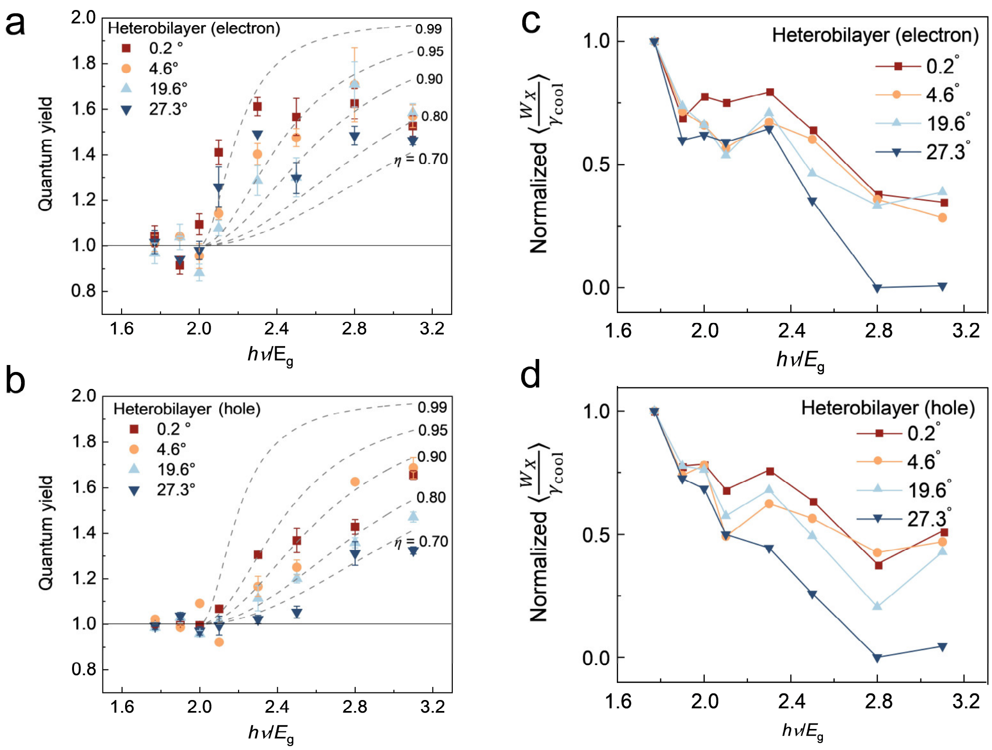

Supplementary Fig. 18 | IXM efficiency vs photon energy. a,b, Twist-angle dependent quantum yield of IX generation for electron and hole states as a function of pump photon energy normalized to the $E_{g(\text{type-II})}$. The dashed lines are calculated IXM efficiency (η) based on Eqn. (1-3) in the model described in Supplementary Note 1. **c,d**, Calculated twist-angle dependence of the scattering rate to cooling rate ratio for electron states as a function of pump photon energy based on Eqn. (4-5) described in Supplementary Note 1.

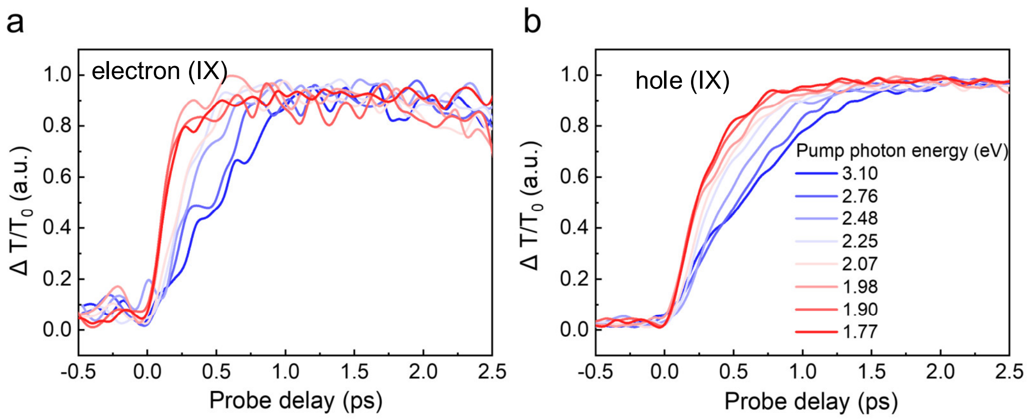

Supplementary Fig. 19 | Rise dynamics. a,b, The normalized photocarriers dynamics probed at MoS_2 (**a**) and WSe_2 (**b**) transitions in a MoS_2/WSe_2 heterobilayer, respectively, exhibit a slower rise with higher excitation energy potentially serving as a distinctive characteristic of IXM.

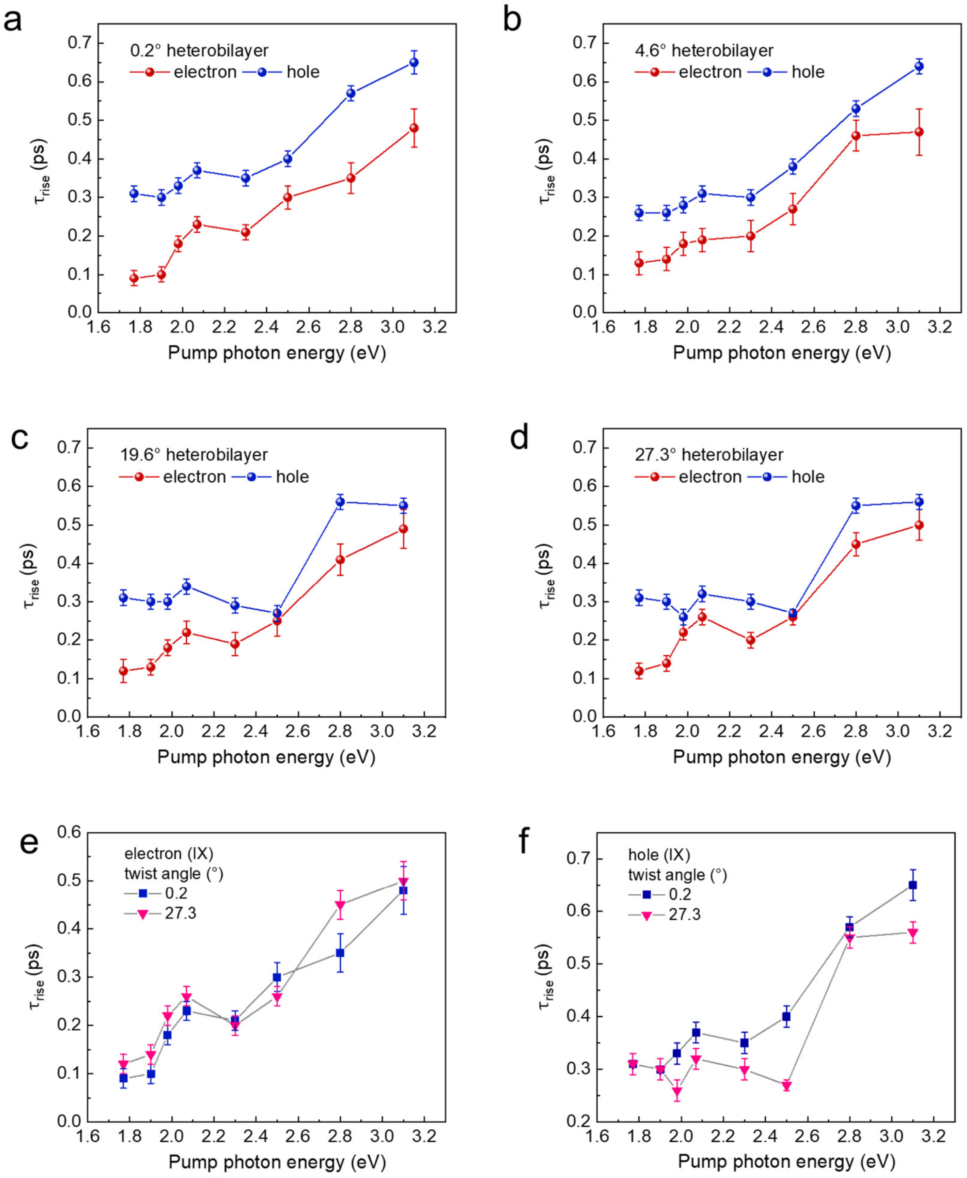

Supplementary Fig. 20 | Fitted rise time. a−d, The rise time acquired by single exponential decay fittings as a function of pump photon energy of in MoS_2/WSe_2 heterobilayer with different twist angles as labeled. **e,f,** The summarized rise time for the electron and hole states of IX as a function of pump photon energy, respectively.

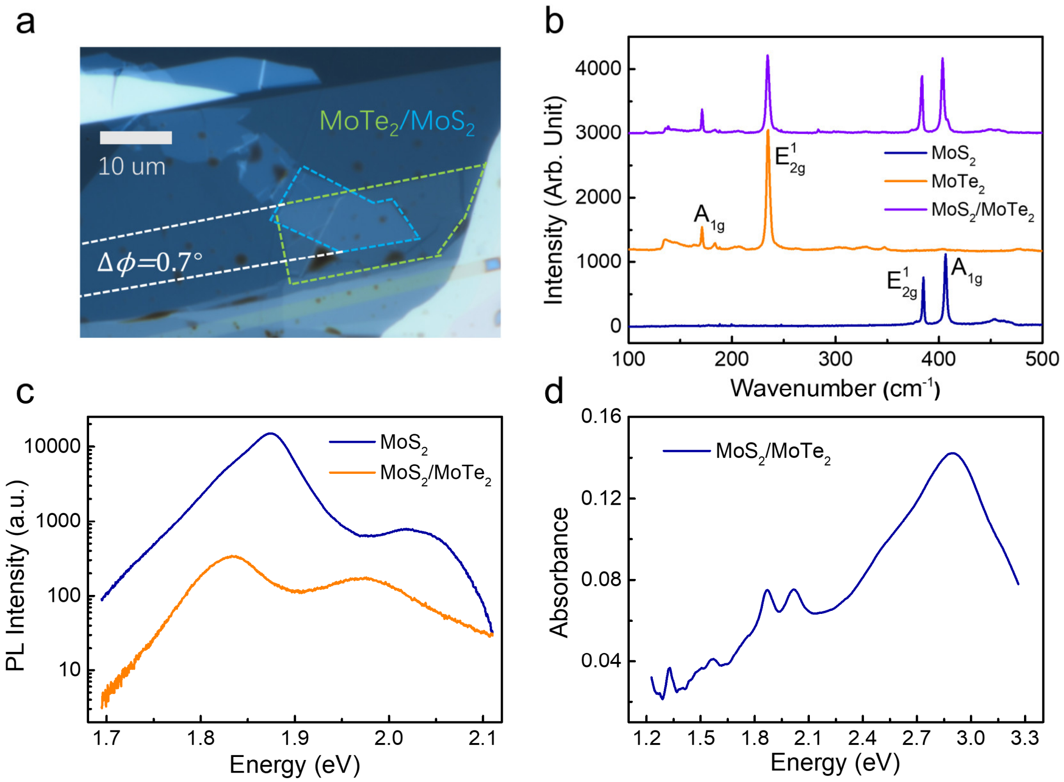

Supplementary Fig. 21 | Optical characterizations of the $MoS_2/MoTe_2$ heterobilayer. a, Optical image of the $MoS_2/MoTe_2$ heterobilayer on a quartz substrate. The twist-angle the layers with $\Delta\phi$ =0.7° (the angle between the white dashed lines) as achieved by aligning the crystallographic axes of the transition metal dichalcogenides (TMDCs) judging by their sharp edges. **b,** Raman spectra of the monolayer $MoTe_2$, monolayer MoS_2, and their heterobilayer samples. The two main peaks E^1_{2g} and A_{1g} for $MoTe_2$ and MoS_2 are assigned to the in-plane and out-of-plane modes, respectively. From the heterobilayer sample, distinct features of both materials are observed. **c,** PL spectra of the $MoS_2/MoTe_2$ heterobilayer and the monolayer MoS_2 samples under the 2.33 eV continuous-wave laser excitation. Such significant PL quenching indicates that efficient interlayer charge transfer. **d,** The absorption spectrum of the $MoS_2/MoTe_2$ heterobilayer is presented for the purpose of calibrating the absorbed photon density at various pump excitation energies.

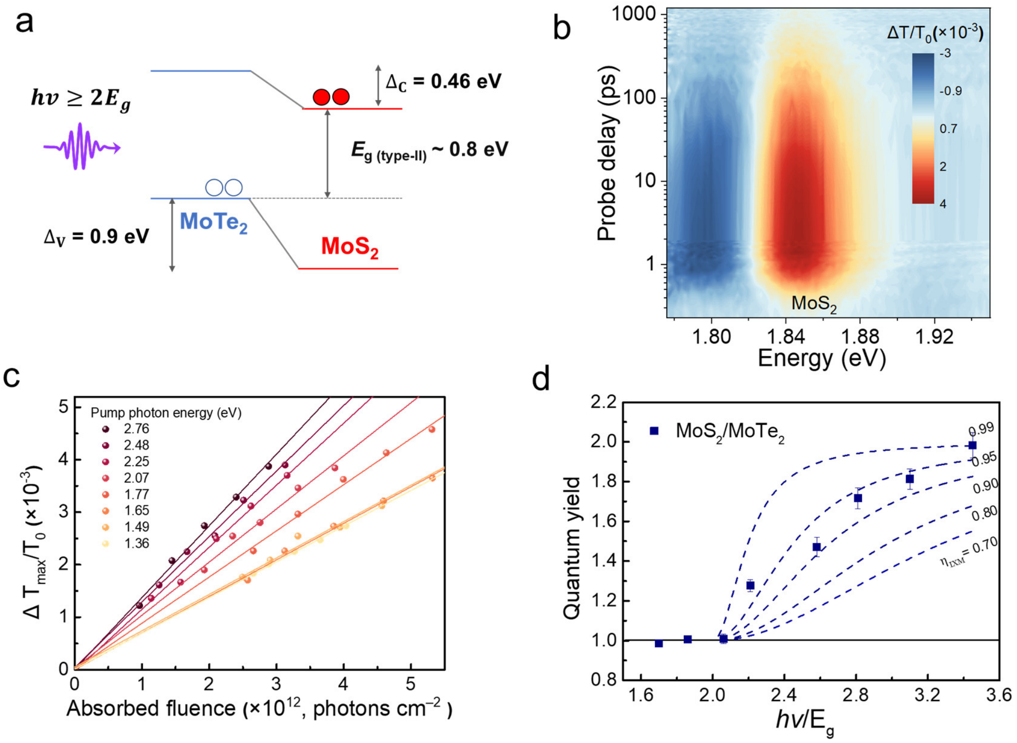

Supplementary Fig. 22 | Interlayer IXM in the $MoS_2/MoTe_2$ heterobilayer. a, Band alignment of the $MoS_2/MoTe_2$ heterobilayer with a type-II band gap of 0.8 eV[24] and band offsets as labeled[25]. **b,** 2D color plot of TA spectra of the $MoS_2/MoTe_2$ heterobilayer excited by a 2.48 eV pump pulse. Probing the MoS_2 band-edge transition enables the characterization of electron states involved in IX dynamics. **c,** The maximum intensity of $\Delta T_{\max}/T_0$ probed at electron states of IX as a function of absorbed photon fluence for varying excitation energies. The solid lines are linear fits. **d,** QY vs pump energy, normalized by the interlayer bandgap (0.8 eV). The dashed lines depict simulation results associated with IXM efficiencies (Supplementary Note 1).

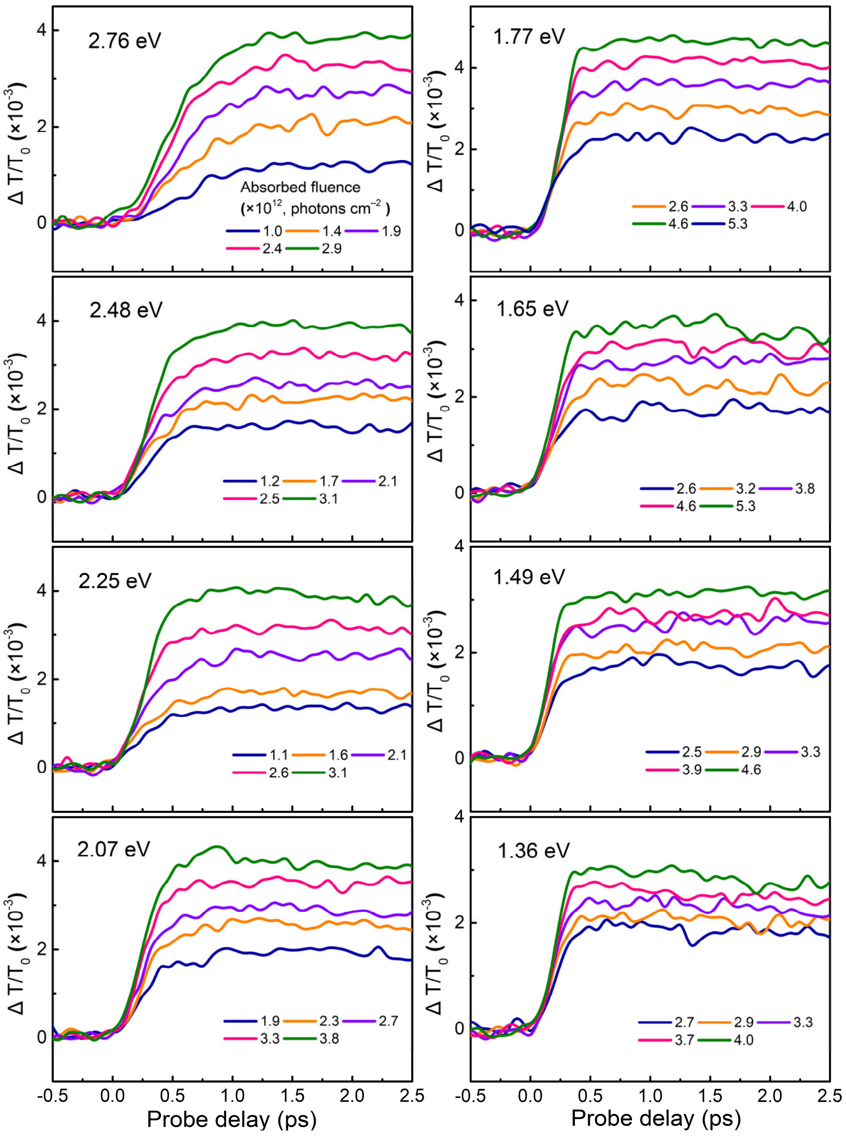

Supplementary Fig. 23 | Absorbed fluence dependence of electron dynamics in IX of the MoS_2/$MoTe_2$ heterobilayer. The probe beam energy is at the MoS_2 band-edge transition to monitor the electron population of IX.

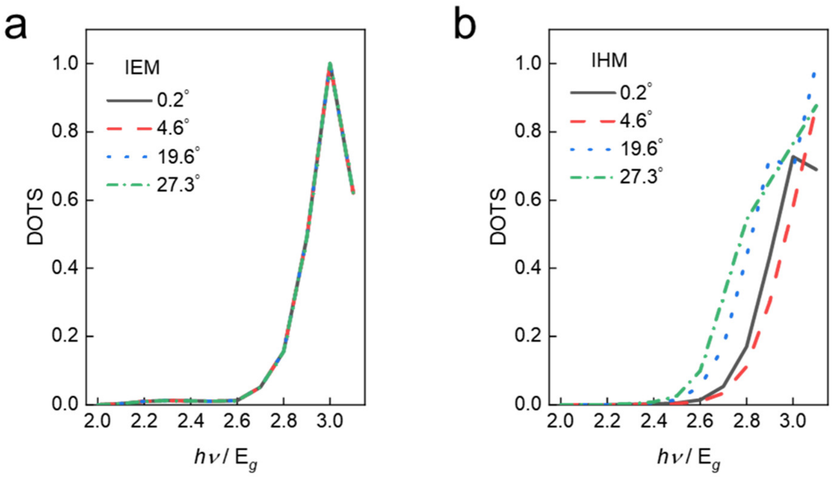

Supplementary Fig. 24 | Density of trion states. a,b, Normalized density of the trion state (DOTS) as a function of photon energy for various twist angles in both the interlayer electron multiplication and interlayer hole multiplication processes.

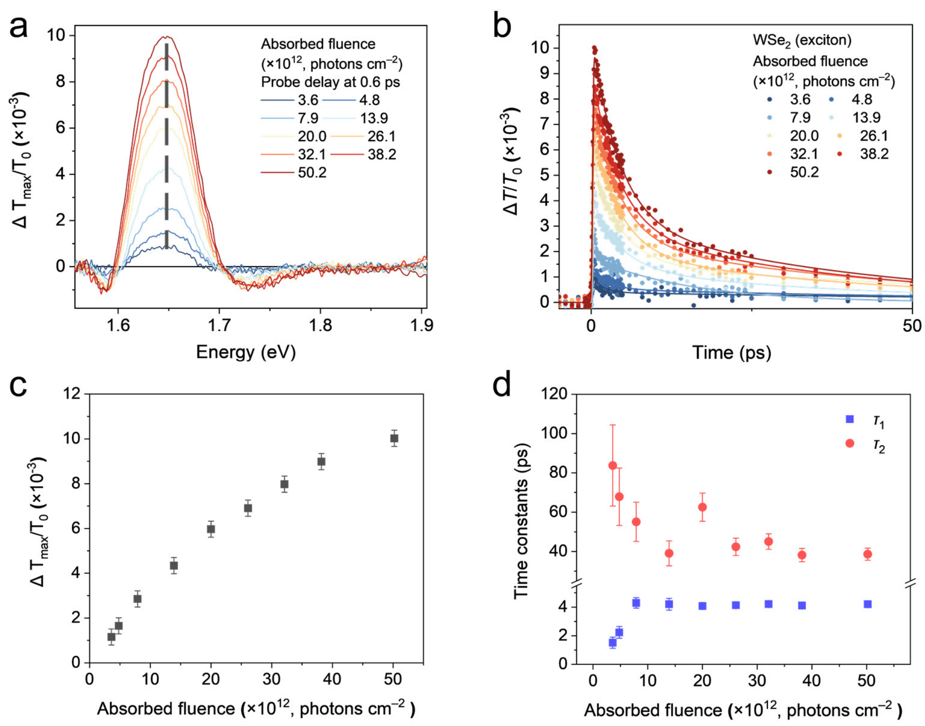

Supplementary Fig. 25 | TA spectroscopy of WSe_2 monolayer under sub-threshold energy excitation conditions with high absorbed fluences. a, TA spectra of the WSe_2 monolayer at the delays of 0.6 ps under various absorbed photon densities as labled. **b**, Absorbed fluence dependence of the exciton dynamics. The curves are bi-exponential fits. **c**, Differential transmittance maximum as a function of the monolayer absorbed photon fluence. **d**, Time constants deduced from the bi-exponential fits shown as the curves in **b** as a function of the absorbed photon fluence.

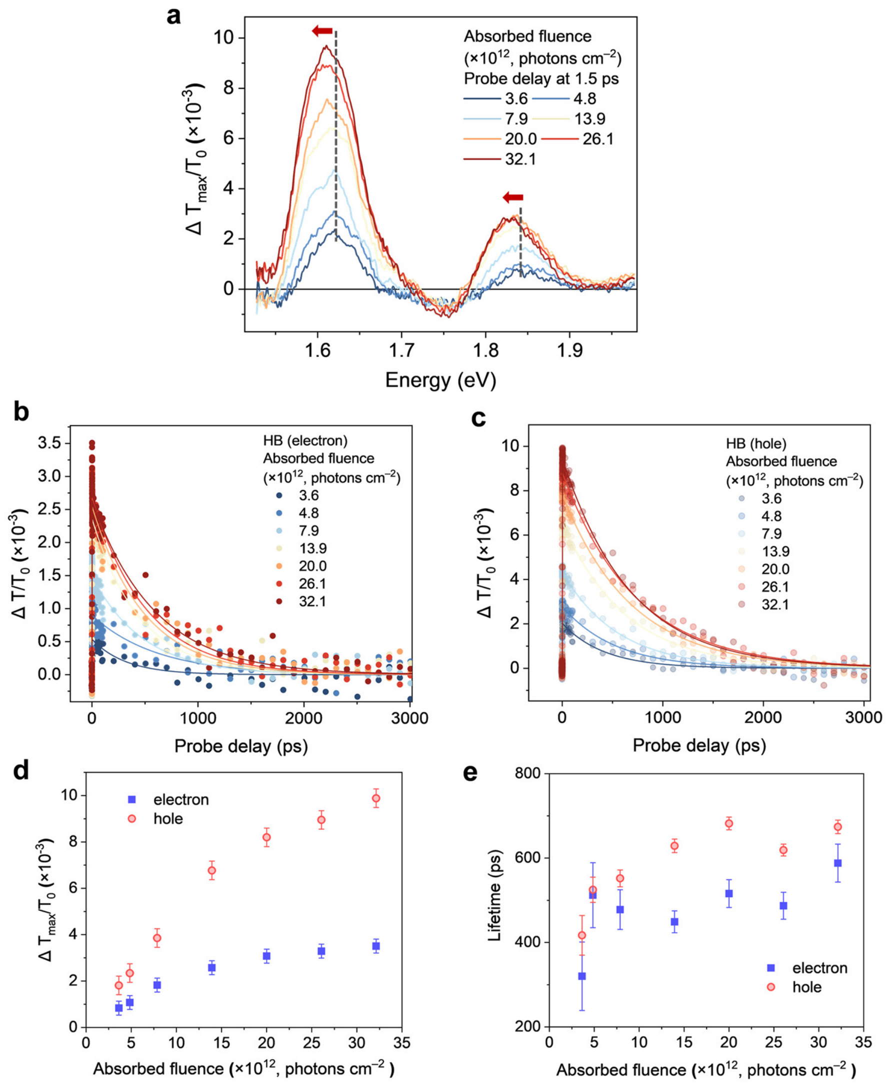

Supplementary Fig. 26 | TA spectroscopy of MoS_2/WSe_2 heterobilayer under sub-threshold energy excitation conditions with high absorbed fluences. a, TA spectra of the 0.2° MoS_2/WSe_2 heterobilayer (HB) at the delays of 1.5 ps under 1.9-eV various absorbed photon densities as labled. **b**,**c**, Absorbed fluence dependence of the electron and hole dynamics of IX states, respectively. The curves are exponential fits. **d**, Differential transmittance maximum as a function of the heterobilayer absorbed photon fluence. **e**, Time constants deduced from the exponential fits shown as the curves in **b** and **c** as a function of the absorbed photon fluence.

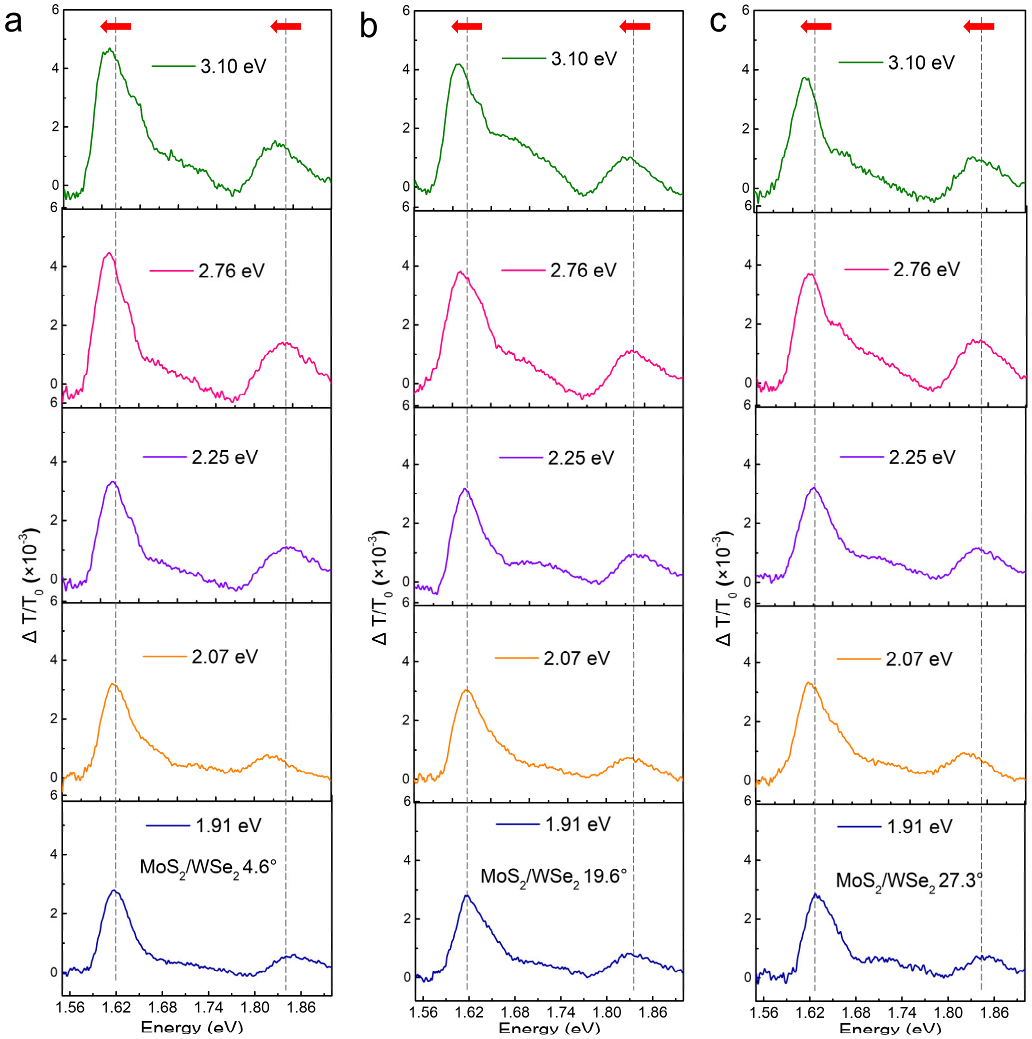

Supplementary Fig. 27 | Redshift of TA peaks. a−c, TA spectra of the MoS_2/WSe_2 heterobilayers with twist angle of 4.6°, 19.6° and 27.3°, respectively, under various the pump photon energies at 1.5 ps with identical injected photocarrier density.

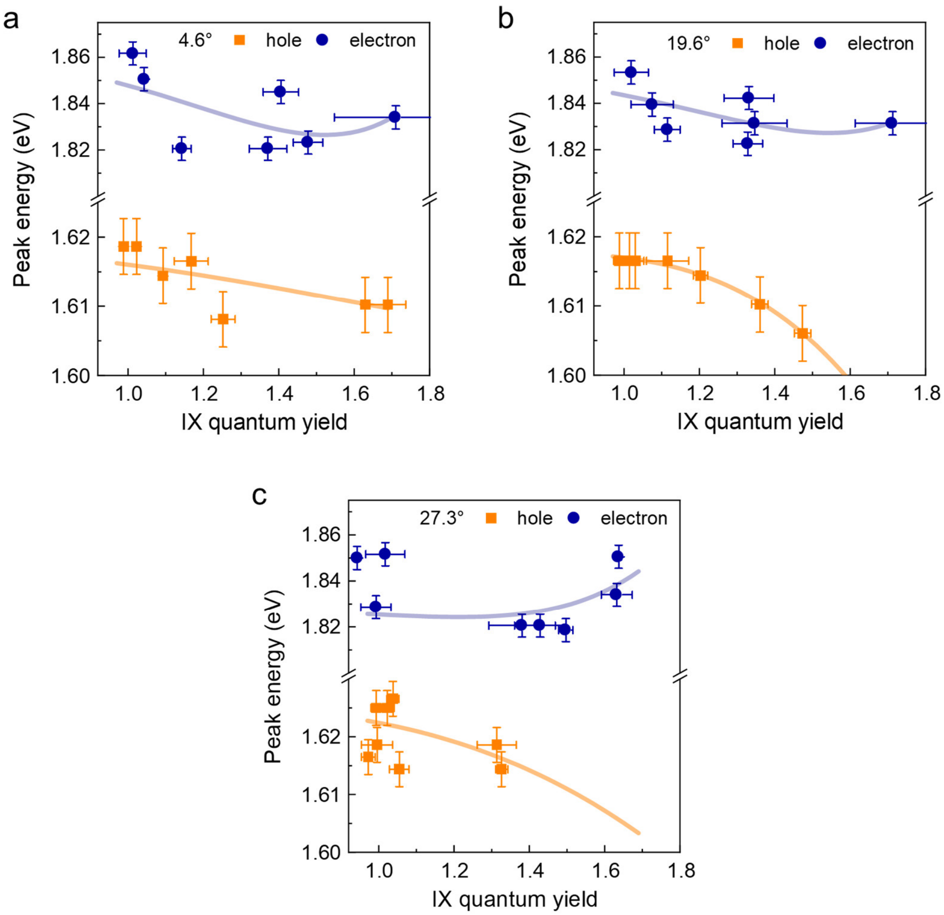

Supplementary Fig. 28 | PB peak energy versus quantum yield. a−c, Peak energy of electron and hole states of IX as a function of carrier quantum yields for heterobilayers with different twist angles as labeled. Solid lines are theoretical calculations.

Supplementary Table 6. Fitted parameters by the Lennard-Jones potential model

Twist angle	0.2°		4.6°		19.6°		27.3°	
	electron	hole	electron	hole	electron	hole	electron	hole
$\boldsymbol{\varepsilon/eV}$	0.13	-0.0013	0.24	0.13	0.18	-0.0090	0.03	-0.0016
$\boldsymbol{r_s}$/nm	4.09	6.22	4.13	3.17	4.10	5.53	4.63	7.20
[m,k]	[12,8]	[8,6]	[12,8]	[8,6]	[12,8]	[8,6]	[12,8]	[6,4]

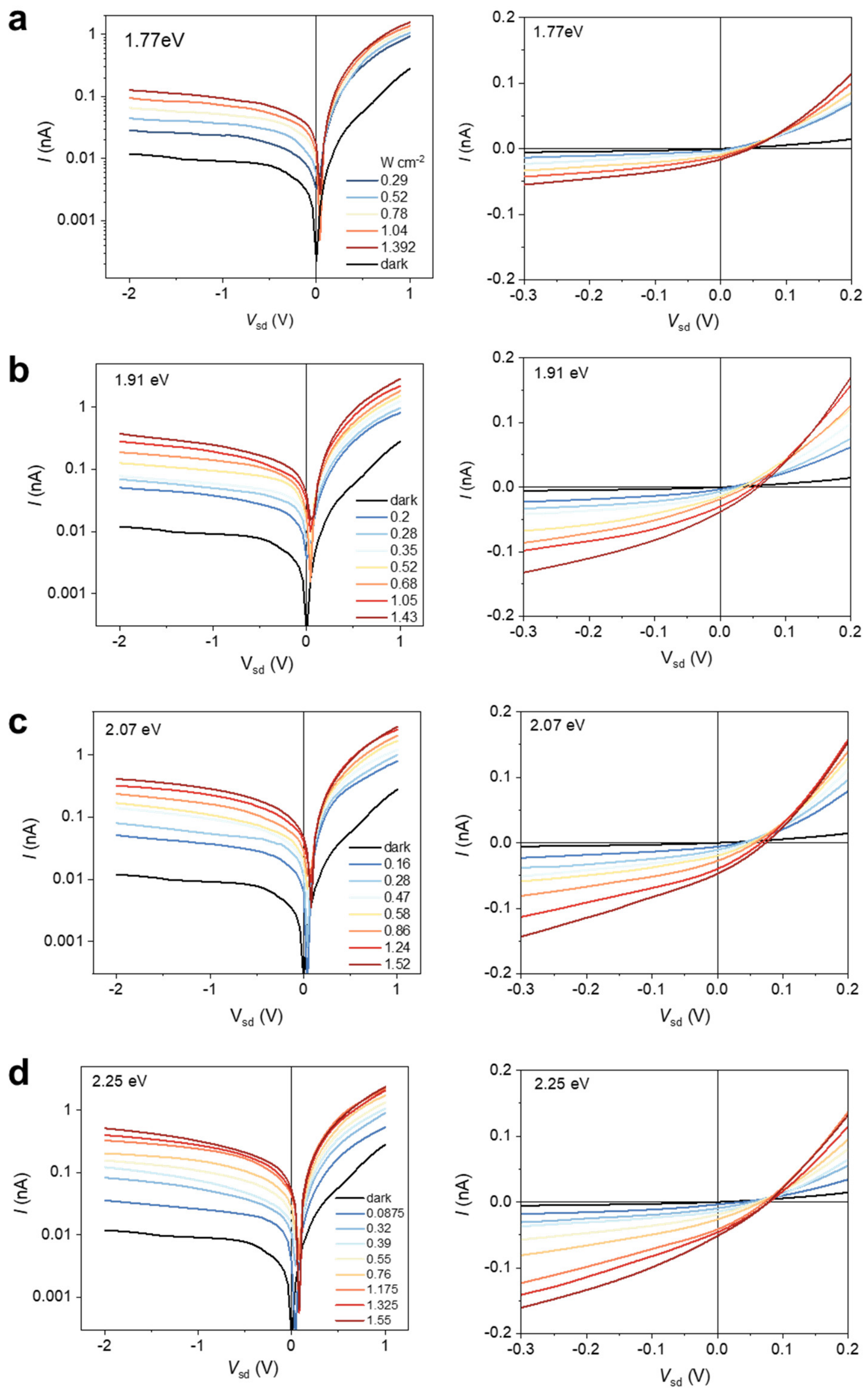

Supplementary Fig. 29 | I−V characteristics under 1.77−2.25 eV. a−d, I−V characteristics of MoS_2/WSe_2 heterobilayer device under monochromatic light illumination with different photon energies and pump intestines as labeled. The right panels are linear plot of I-V curves near 0V.

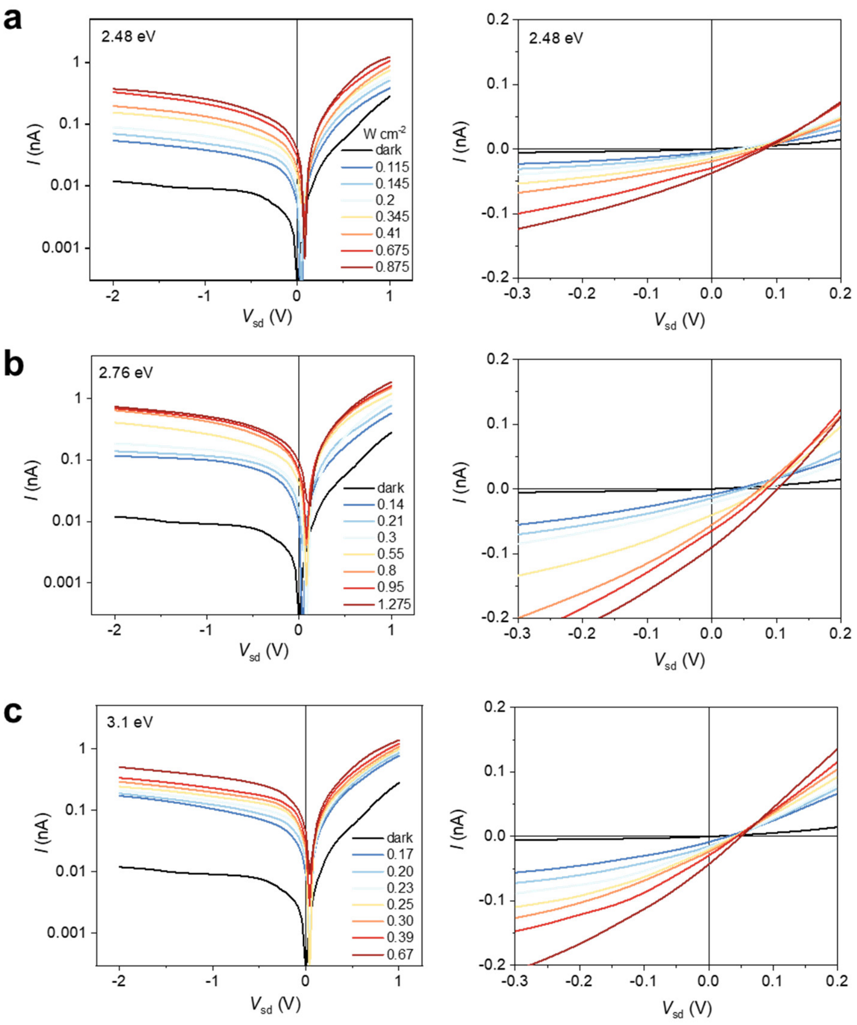

Supplementary Fig. 30 | I−V characteristics under 2.48−3.10 eV. a−c, I−V characteristics of MoS_2/WSe_2 heterobilayer device under monochromatic light illumination with different photon energies and pump intestines as labeled. The right panels are linear plot of I-V curves near 0V.

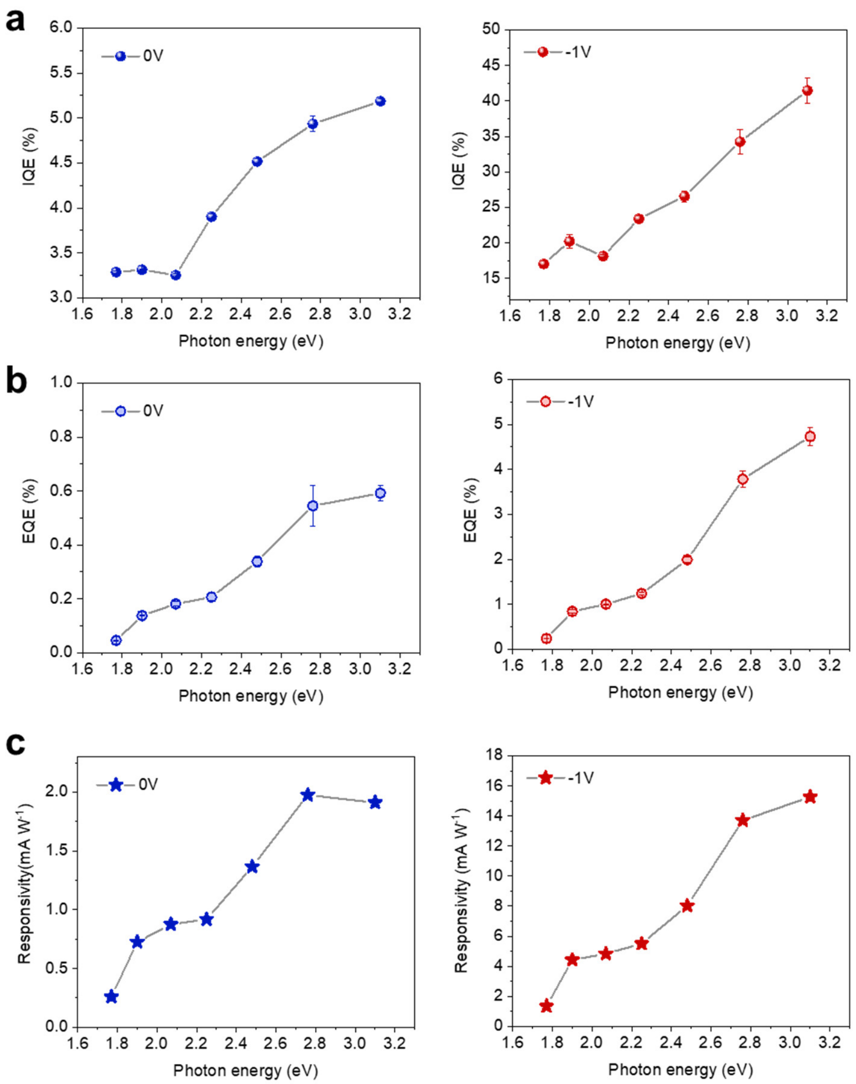

Supplementary Fig. 31 | IQE, EQE and responsivity. a−c, Internal quantum efficiency (IQE), external quantum efficiency (EQE) and responsivity of MoS_2/WSe_2 heterobilayer device as a function of pump photon energies under 0V (left) and -1V bias (right).

Supplementary References

1. Beard, M.C. et al. Comparing multiple exciton generation in quantum dots to impact ionization in bulk semiconductors: implications for enhancement of solar energy conversion. *Nano Letters*. **10**, 3019-3027 (2010).
2. Luo X*, et al.* Effects of lower symmetry and dimensionality on Raman spectra in two-dimensional WSe_2. *Physical Review B* **88**, 195313 (2013).
3. Li H*, et al.* From bulk to monolayer MoS_2: evolution of Raman scattering. *Advanced Functional Materials* **22**, 1385-1390 (2012).
4. Yuan Y*, et al.* Probing the Twist-Controlled Interlayer Coupling in Artificially Stacked Transition Metal Dichalcogenide Bilayers by Second-Harmonic Generation. *ACS Nano* **17**, 17897-17907 (2023).
5. Nayak PK*, et al.* Probing evolution of twist-angle-dependent interlayer excitons in $MoSe_2/WSe_2$ van der Waals heterostructures. *ACS Nano* **11**, 4041-4050 (2017).
6. Chiu M-H*, et al.* Spectroscopic signatures for interlayer coupling in MoS_2–WSe_2 van der Waals stacking. *ACS Nano* **8**, 9649-9656 (2014).
7. Li Y*, et al.* Measurement of the optical dielectric function of monolayer transition-metal dichalcogenides: MoS_2, $MoSe_2$, WS_2, and WSe_2. *Physical Review B* **90**, 205422 (2014).
8. Qiu DY, Da Jornada FH, Louie SG. Optical spectrum of MoS_2: many-body effects and diversity of exciton states. *Physical Review Letters* **111**, 216805 (2013).
9. Fan X, Singh DJ, Zheng W. Valence band splitting on multilayer MoS_2: mixing of spin–orbit coupling and interlayer coupling. *Journal of Physical Chemistry Letters* **7**, 2175-2181 (2016).
10. Zhang Y, Li H, Wang H, Liu R, Zhang S-L, Qiu Z-J. On valence-band splitting in layered MoS_2. *ACS Nano* **9**, 8514-8519 (2015).
11. Carvalho A, Ribeiro R, Neto AC. Band nesting and the optical response of two-dimensional semiconducting transition metal dichalcogenides. *Physical Review B* **88**, 115205 (2013).
12. Debbichi L, Eriksson O, Lebègue S. Electronic structure of two-dimensional transition metal dichalcogenide bilayers from ab initio theory. *Physical Review B* **89**, 205311 (2014).
13. Yue Y-Y*, et al.* Many-particle induced band renormalization processes in few-and mono-layer MoS_2. *Nanotechnology* **32**, 135208 (2021).
14. Bieniek M, Szulakowska L, Hawrylak P. Band nesting and exciton spectrum in monolayer MoS_2. *Physical Review B* **101**, 125423 (2020).
15. Rigosi AF, Hill HM, Li Y, Chernikov A, Heinz TF. Probing interlayer interactions in transition metal dichalcogenide heterostructures by optical spectroscopy: MoS_2/WS_2 and $MoSe_2/WSe_2$. *Nano Letters* **15**, 5033-5038 (2015).
16. Cui Q, Ceballos F, Kumar N, Zhao H. Transient Absorption Microscopy of Monolayer and Bulk WSe_2. *ACS Nano* **8**, 2970-2976 (2014).
17. Nie Z*, et al.* Ultrafast carrier thermalization and cooling dynamics in few-layer MoS_2. *ACS Nano* **8**, 10931-10940 (2014).

18. Ceballos F, Cui Q, Bellus MZ, Zhao H. Exciton formation in monolayer transition metal dichalcogenides. *Nanoscale* **8**, 11681-11688 (2016).
19. Steinleitner P*, et al.* Direct Observation of Ultrafast Exciton Formation in a Monolayer of WSe_2. *Nano Letters* **17**, 1455-1460 (2017).
20. Valencia-Acuna P, Zereshki P, Tavakoli MM, Park J-H, Kong J, Zhao H. Transient absorption of transition metal dichalcogenide monolayers studied by a photodope-pump-probe technique. *Physical Review B* **102**, 035414 (2020).
21. Wang H, Zhang C, Rana F. Ultrafast dynamics of defect-assisted electron–hole recombination in monolayer MoS_2. *Nano Letters* **15**, 339-345 (2015).
22. Shockley W, Queisser HJ. Detailed balance limit of efficiency of p-n junction solar cells. *Journal of Applied Physics***32**, 510-519 (1961).
23. Rühle S. Tabulated values of the Shockley–Queisser limit for single junction solar cells. *Solar Energy***130**, 139-147 (2016).
24. Ju Q*, et al.* Infrared Interlayer Excitons in Twist-Free $MoTe_2/MoS_2$ Heterobilayers. *Advanced Materials***36**, 2404371 (2024).
25. Quan C*, et al.* Band alignment of $MoTe_2/MoS_2$ nanocomposite films for enhanced nonlinear optical performance. *Advanced Materials Interfaces***6**, 1801733 (2019).
